# The Child and Adolescent Thriving Index 1.0: Developing a Measure of the Outcome Indicators of Well-Being for Population Health Assessment

**DOI:** 10.1007/s12187-022-09962-0

**Published:** 2022-08-06

**Authors:** Nathaniel W. Anderson, Anna J. Markowitz, Daniel Eisenberg, Neal Halfon, Kristin Anderson Moore, Frederick J. Zimmerman

**Affiliations:** 1 Department of Health Policy and Management, UCLA, Los Angeles, CA USA; 2 Department of Education, UCLA, Los Angeles, CA USA; 3 Department of Health Policy and Management, Department of Pediatrics, UCLA, Los Angeles, CA USA; 4 Child Trends, Bethesda, MD USA

**Keywords:** Child Well-Being, Adolescent Well-Being, Measurement, Composite Indices, Population Health, Predictive Validity

## Abstract

The well-being of children and adolescents is emerging as an area of interest for population health measurement. Previous approaches assessing national and state trends in well-being have relied on composite indices. However, these methodologies suffer from several weaknesses. This paper develops an improved index for the United States that is measurable with existing population-data resources. It derives the appropriate weights for items in this index using a longitudinal panel of 2,942 children in the Panel Study of Income Dynamics. Candidate component measures are selected for the index based on their demonstrated association with several subjective scales assessed during young adulthood. The final index demonstrates that a broad range of measures indicate higher levels of population-level well-being. The predictive validity of the index for outcomes during young adulthood is also assessed: a one-standard-deviation increase in the index score is associated with a 7.9-percentage-point decrease [95% CI: 5.9 – 9.8] in ever reporting fair or poor health, a 6.3-percentage-point decrease [95% CI: 4.6 – 8.0] in ever reporting depression, and a 17.2% [95% CI: 13.7% – 20.5%] increase in peak earnings. These values for predictive validity are slightly higher than those of existing methodologies. We also find that incorporating contextual indicators from childhood and adolescence does not substantively improve predictive validity. Policy-makers and government agencies interested in population-level well-being of children and adolescents can continue to use existing indices as reasonable proxies, but should also commit to upgrading data systems to make them more child-centric in the future.

**Supplementary Information:**

The online version contains supplementary material available at 10.1007/s12187-022-09962-0.

## Introduction

In recent years, public health leaders in the United States have placed greater emphasis on well-being as a key indicator of population health. Despite this increased interest, its measurement remains underdeveloped. Healthy People 2030 explicitly highlights well-being as one of its eight overall key measures, yet it is the only one for which there is no agreed-upon system of measurement (Healthy People 2030, 2020). There is accordingly substantial need for the development of rigorous, concrete, and easily constructed measures of well-being.

Childhood and adolescence are particularly important stages in the lifecourse with regards to well-being (Ben-Arieh, 2008; Handbook of Child Well-Being, 2014). Numerous indices have emerged with the goal of capturing the well-being of children and adolescents in the United States (Buck et al., 2018; Land et al., 2001, 2007; Moore et al., 2007, 2008; O’Hare & Bramstedt, 2003; O’Hare et al., 2013). However, these measures suffer from several issues that prevent more widespread usage. First, the methodologies behind these indices have not been validated against important outcomes at the individual level. Second, index components tend to comprise both individual outcomes and contextual determinants of well-being. While improvements in both of these categories correspond to enhanced life prospects for children and adolescents, the mixing of individual and contextual variables within existing well-being indices risks conflating variables at different points in a causal pathway and therefore weakens them as tools for scientific inquiry (Moore et al., 2007). Third, measurement of well-being in the United States does not reflect children and adolescents’ subjective perception of their own well-being (Bardo, 2017; Bradshaw, 2015; Casas, 2011), even though it is likely critical in identifying subtle but important issues that are missed by the existing set of standard proxy indicators.

This paper utilizes a longitudinal panel to develop an index that addresses several of these concerns while remaining compatible with the data systems currently available for reconstructing them at the population-level. The primary aim of this work is to develop an index for population-level monitoring of well-being, and to assess its predictive validity for future health and earnings during young adulthood.

The secondary aims are threefold: 1) to assess whether the predictive validity of this improved index is meaningfully better than one of the main indices currently in use in the United States; 2) to assess whether an index restricted to using only individual outcomes as components performs similarly to one that includes contextual measures; and 3) to assess whether adopting subjective scales to benchmark the index leads to quantifiable improvements in predictive validity.

## Background

There are numerous approaches to defining well-being across the disciplines of health, psychology, sociology, economics, and others (Handbook of Child Well-Being, 2014). A recent Robert Wood Johnson Foundation conference offers a compelling definition from a public-health perspective:

“*the comprehensive view of how individuals and communities experience and evaluate their lives, including their physical and mental health and having the skills and opportunities to construct meaningful futures”* (Robert Wood Johnson Foundation, 2019).

Under this framework, the “being” part of well-being is representative of two different, but related, concepts: the present state of “being,” and the future-oriented process of “becoming” (Ben-Arieh & Frones, 2011). Measures of child and adolescent well-being seek to balance the current status of youth, such as their happiness and relationships, with their potential to thrive in the future, such as their educational prospects. Many aspects serve both roles, such as their health and economic conditions. In recent years, increasing attention has been paid to the importance of including measures of positive mental health, which capture facets of well-being beyond the traditionally emphasized measures of mental distress (Wiese et al., 2018).

Numerous competing approaches to defining well-being have emerged, posing additional challenges for measurement. For example, various philosophers have tried to devise lists of objective measures that are necessary to achieve a life of meaning and well-being (Adler & Fleurbaey, 2016; Nussbaum, 2009), while researchers in psychology have stressed the importance of the subjective experience, which emphasizes how high individuals self-evaluate their quality of life (Diener et al., 2018). Within discussions of subjective well-being, there is further debate between the *hedonic* tradition, which emphasizes measures of emotion and feeling such as happiness and life satisfaction, and the *eudaimonic* tradition, which focuses on agency and capabilities such as personal growth and self-actualization (Ryan & Deci, 2001). Adjudicating between these perspectives is challenging, and in recent years consensus has formed that both perspectives contribute valuable insight as to our understanding of well-being (Diener et al., 2018). Recent empirical work has supported this by showing both constructs can be distinctly identified within individuals (Henderson & Knight, 2012; Mishra et al., 2019). Thus, to the extent possible, we believe population-level measurement of well-being should incorporate insights from each tradition, while carefully acknowledging the limitations in methodology and data-availability that cause disconnects between theory and quantitative assessment.

Since well-being is a complex and multidimensional construct, it cannot be represented by a single survey item (Benjamin et al., 2020). Furthermore, in the United States, subjective well-being data collection for nationally representative samples is fairly limited. Therefore, for the purposes of monitoring at the population-level, researchers typically operationalize well-being by first gathering data across a defined set of component measures and then aggregating the information into a single estimate in the form of an index. Potential components (e.g. poverty, mortality, morbidity, educational attainment, etc.) are selected so as to embody the broad definition offered above, while also remaining simple enough to be useful as a tool for monitoring and evaluation. Since these components are not direct measures of well-being, but rather a set of proxy measures, we refer to them as *outcome indicators* of child and adolescent well-being, as suggested by previous researchers (Ben-Arieh & Frones, 2011).

Numerous indices comprised of outcome indicators of child and adolescent well-being have been developed in recent decades. In the United States the most prominent are the Child and Youth Well-Being and the KIDS COUNT indices (Land et al., 2001, 2007; O’Hare & Bramstedt, 2003; O’Hare et al., 2013). However, these indices have certain limitations that may prove significant. First, there are a set of methodological criticisms concerned with whether these indices accurately evaluate well-being. Chief among these is that components are selected solely on the basis of expert-informed opinion, and often contribute equally to the overall summary measure. Although this approach has inspired important debates as to how measures are chosen and aggregated (Hagerty & Land, 2007; Lippman et al., 2011; Moore et al., 2008), no study has yet used a quantitative approach to resolve these issues.

Second, when selecting component measures to represent well-being, existing methodologies tend to conflate individual-level outcomes with their contextual determinants (Moore et al., 2007, 2008). By individual-level level outcomes, we mean measures that correspond to the status of an individual child or adolescent, such as their academic achievement, health status, or health behaviors. By contextual determinants, we mean measures that evaluate the surrounding environment children live in, such as household income or employment status of their parent/guardian. These two classes of measures are mixed together in part due to differences in aims and/or intended audiences: including contextual determinants in an index more oriented towards advocacy and/or the general public may be beneficial for rallying popular support. However, if a key goal of the development of a child and adolescent well-being index is to understand how systems and policies affect youth, then contextual determinants should not be included (Moore et al., 2014). Put another way, these factors, which conceptually are *causes* of individual-level differences in well-being, should not also be included in the measure of the *effect*.

Lastly, existing indices are not grounded in children and adolescents’ own assessment of their subjective well-being. Instead, they rely entirely on statistics already collected by government agencies, institutions that may have their own preconceived notions as to what is most important (Jenkins, 2019). As mentioned, well-being researchers have argued convincingly that incorporating the subjective perspective into indices is necessary from a rights perspective (Ben-Arieh et al., 2014). This sort of recognition may also be directly beneficial to children and adolescents: successfully shifting cultural practices to value their present needs alongside their future aspirations may have beneficial effects on their mental health (De Mol et al., 2018). Furthermore, it seems likely that a rebalancing that places greater emphasis on subjective measures would improve accuracy of well-being measurement (Axford et al., 2014; Bradshaw, 2019).

## Methods

### Data

We use the Panel Study of Income Dynamics (PSID), a household panel study maintained by the Institute for Social Research at the University of Michigan (Panel Study of Income Dynamics, 2021). We view the PSID as the best available data source for our research aim, since to our knowledge is the only source of data of its size and methodological rigor that collects a rich array of measures affecting and representing health and well-being of American children and adolescents, including several direct measures of subjective well-being. We use the weights provided in the PSID to account for attrition in the final regression models, as well as adjusting for complex survey design. This study was approved by the Institutional Review Board at a large university in the United States, which waived the need for informed consent due to the use of deidentified data.

### Sample

In 1997, the PSID fielded its first Child Development Supplement (CDS) collecting information for up to two children aged 0–12 within participating families, with the intention of developing a nationally-representative longitudinal database to study the process of early human capital formation. The measures we use from the CDS include data collected directly from children, as well as from their parent/guardian. Ultimately, 3,563 children participated in the first CDS. Our final analytic sample consists of 2,942 (82.6%) children who subsequently completed at least one of the Transition to Adulthood Supplements (TAS) between ages 18 and 28 during the years 2005–2017. Appendix Fig. 2 contains more information on how the final analytic sample progressed through waves of the survey.

### Subjective Measures

The TAS collects several subjective scales that can serve as the observed approximation of the latent construct of well-being. Rather than select a single one as the best representation of well-being, we analyze each outcome measure separately and then aggregate the results into a single measure. The five scales, all assessed in young adulthood, ages 18–28, are:

- *Flourishing*, constructed from a set of 14 questions adopted from the MacArthur Foundation’s Midlife in the United States Survey (Keyes, 2002).
- *Kessler’s K-6 Non-Specific Psychological Distress*, constructed from a series of six questions assessing their experiences in the past month (Kessler et al., 2003).
- *Economic worry*, constructed from a series of three questions assessing how many days a week on average a person worries about being able to afford things, finding a job, and their overall future prospects.
- *Social Anxiety*, constructed from a series of six questions assessing how many days a week on average a person feels distress from social situations.
- *Life Satisfaction*, taken from a single question asked on a 5-point scale.

Within the field of subjective well-being research, flourishing and life satisfaction are standard measures commonly found in the literature (Diener et al., 2018), while the others are conceived as related to positive functioning, which is a subcategory of subjective well-being measures regarding how individuals express themselves and operate within the world to achieve a sense of higher purpose and achievement (Magyar & Keyes, 2019). Although these three scales are not traditional measures of subjective well-being, they do have demonstrated associations with more standard subjective well-being measures in other analyses (De Castella et al., 2014; Öztürk & Mutlu, 2010; Roth et al., 2017; Russell & Topham, 2012; Winefield et al., 2012). Furthermore, each of the scales has been extensively validated in their own right (Arocho & Purtell, 2020; Bandelj & Lanuza, 2018; Diener et al., 2018; Kessler et al., 2003; Keyes, 2006a, b; Keyes, 2006a, b; Lehnart et al., 2010), and several have well-documented longitudinal associations with improved physical and mental health outcomes (Diener et al., 2017), including mortality [flourishing: (Keyes & Simoes, 2012; Louie et al., 2021); psychological distress: (Yang et al., 2020); life satisfaction: (Kim et al., 2021; Martín-María et al., 2017)], cardiovascular disease [psychological distress: (McLachlan & Gale, 2018); life satisfaction: (Boehm et al., 2011, 2016; Feller et al., 2013)], and mental illness [flourishing: (Keyes et al., 2010); economic worry: (Meltzer et al., 2013; Richardson et al., 2013; Shek, 2003); social anxiety: (Beesdo et al., 2007)]. Appendix Table 6 shows the component questions for each scale. Since they are potentially collected multiple times during young adulthood and on different point scales, they are averaged within individual across young-adulthood waves and then standardized.

### Candidate Index Components

The PSID also collects a series of health, economic, education, and other measures from sample children and their families. Among these measures, we consider those that have data available at the population-level in the United States as candidate components for an index of the outcome indicators of child and adolescent well-being (Appendix Table 7). By the term *candidate*, we mean these measures are *considered* for inclusion in the final index based on the strength of their association with at least one of the five subjective outcomes, which is described in the next section.

Based on the recommendation from Moore and colleagues (Moore et al., 2007, 2008, 2014), we divide the components into two groups: individual-level components and contextual components (defined above). Our main index specification uses only individual-level components, while one of the secondary analyses compares this with an index that uses both individual-level and contextual components. As previously mentioned, we make this distinction since contextual measures are *always* causal factors of well-being, meaning they should not be considered as an outcome indicator of well-being. While individual-level variables may sometimes act as causal factors – for example, healthy behaviors such as smoking and drinking could lead to future poor well-being – there is enough ambiguity about the directionality of the relationship that these individual-level variables could also be considered as a manifestation of existing levels of well-being – for example, smoking or drinking could be a consequence for youth already experiencing low subjective well-being.

Approximately 40% of the sample has at least one missing outcome or covariate. We apply multiple-imputation techniques in order to retain the entire sample. Using chained equations, 20 imputations are calculated using STATA’s *mi impute chained* suite of commands (White et al., 2011). Appendix Table 8 shows descriptive statistics related to the imputation process. The difference in the descriptive samples across the samples with and without the multiple imputation procedure confirms that the missingness is not completely at random, thereby validating our usage of the method.

### Model Selection Procedure for Identifying Final Set of Index Components

Our overall approach is to regress the directly-measured subjective outcomes on a relatively sparse set of component measures that are routinely measured in the general population. This approach has been classified as *hedonic* weighting (Decancq & Lugo, 2013), whereby components are weighted based on their implicit relationship to a measure of subjective well-being. This latent-model estimation allows us to create a more child-centric index of well-being that is constructed from variables commonly available, thereby enabling future monitoring of child and adolescent well-being at the population-level.

First, to achieve some degree of parsimony in the final index, we implement a model selection process to remove candidate components that are weakly related to the subjective outcomes. This model selection process needs to accommodate multiple imputation and attrition weights, as well as provide standard error estimates so that future work can assess uncertainty when applying the index to population-level data.

Our model-selection approach relies heavily on a machine learning statistical technique called Least Absolute Shrinkage and Selection Operator (LASSO) that modifies original least squares by adding penalty parameters to the sum of squared residuals. This has the effect of forcing the coefficients of less significant regressors to drop to 0, making it a popular data-driven method for model selection. The value of the penalty is determined through cross-validation – we use an 80/20 split between training and testing data. We use the adaptive LASSO algorithm, a modification of the original LASSO procedure that improves upon its consistency.

The procedure for combining LASSO with multiple imputation is not widely agreed upon. Averaging the results is not ideal for achieving the desired result of a more parsimonious model, since a covariate that was kept for 1 of the 20 imputations would ultimately be included in the final model. Additionally, LASSO techniques were not developed to be used with complex survey data adjusting for attrition. There is some recent work that has modified LASSO and other model selection techniques to these sorts of datasets (McConville, 2011; Wang et al., 2014). However, to our knowledge there has not been work using these methods with multiply-imputed data.

Our overall approach is a two-step process based on the recommendations of a simulation analysis (Wood et al., 2008). The first step is to run the adaptive LASSO procedure without the attrition weights for each of the 20 imputations and initially retain component variables kept during 10 or more of the imputations. For the second step, we run a series of standard linear regression with the attrition weights. After each regression, we manually remove component variables with highly nonsignificant p-values (> 0.25) one-by-one (see Appendix Table 9 for more details about this process). These are variables kept by the adaptive LASSO because they improve the prediction of the model despite a lack of precision for the coefficient estimate, or because the attrition weights strongly affect the results. Additionally, some coefficients may have signs in the opposite direction as anticipated. One example is teen pregnancy, a measure that has well-demonstrated associations with adverse socioeconomic and health outcomes, but is associated with higher levels of life satisfaction in our analysis. Since we believe the index would be difficult to interpret in these instances, we opt to remove the component from the preferred index specification to keep the values of the weights more intuitive for a policy audience.

We perform this two-step process (Adaptive LASSO, then backwards selection) five times, once for each subjective outcome, and control for several demographic measures (average age [continuous], sex [male and female], and race/ethnicity [white, Black, Latinx, and Other]) throughout the procedure.

There is concern that running a linear model post-LASSO raises issues similar those encountered in stepwise regression, in that the procedure may lead to unrealistically small standard error estimates, although some have argued that this procedure can be run under a restrictive set of assumptions (Zhao et al., 2021). However, there is not yet a consensus for estimating standard errors of covariates kept during the LASSO procedure (Kyung et al., 2010). In the absence of a clear alternative, we adopt the approach laid out here with the belief that this imperfect procedure should be less biased than those assuming equal weighting of components chosen on the basis of expert opinion. To illustrate that the point estimates of final components are not heavily influenced by the finer points of this model selection process, Appendix Table 10 shows a side-by-side comparison of key coefficients before and after the model selection procedure.

### Deriving Final Index Weights

**Fig. 1:**
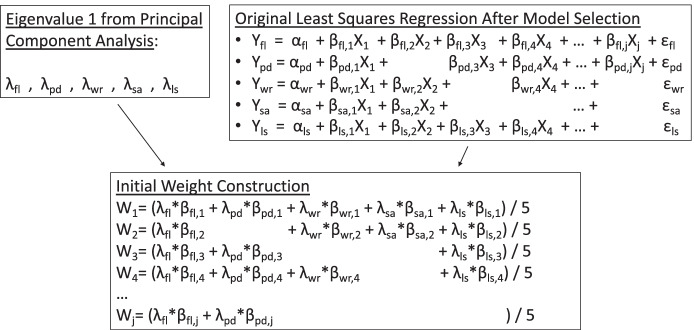
Visual Representation of Index Weight Derivation After Model Selection Procedure. Notes: Arrows show progression through the index derivation process. fl = flourishing; pd = non-specific psychological distress; wr = economic worry; sa = social anxiety; ls = life satisfaction. λ = factor loadings from first eigenvalue of Principal Component Analysis (PCA). X_i_ = *i*th observed component (varies in each regression equation based on model selection. j = last component surviving model selection. W_i_ = Nonstandardized index weight of *i*th component

Once the set of regression equations is finalized for each of the five subjective outcomes, we are able to derive the final index weights through a three-step process (Fig. 1 provides a visual representation). First, we run ordinary least squares regression of the subjective outcome on the components that survive the model selection procedure, plus demographic variables. This is done five times, once for each subjective outcome and its unique set of respective component measures, leaving us with five vectors of component coefficients. Components removed during the model selection process are assumed to have a coefficient value of 0 in that regression equation.

Second, we separately conduct a principal component analysis (PCA) of the five subjective outcomes. Principal component analysis is a commonly-used technique for reducing the dimensions of several correlated measures while accounting for the maximum possible variation among the original measures. It has been used in other research of indices of well-being to directly derive component weights (Knippenberg, 2017; Ogwang & Abdou, 2003), but for our purposes it serves as a useful tool for aggregating across the five vectors of regression coefficients.

For the third step, we combine the results from the prior two steps to construct index weights using a double weighting system. Equation 1) illustrates this strategy for a single component weight:

- $${CompWght}_{z}=(\left({PCA}_{fl}*{\beta }_{fl,z}\right)+\left({PCA}_{pd}*{\beta }_{pd,z}\right)+\left({PCA}_{wr}*{\beta }_{wr,z}\right)+\left({PCA}_{sa}*{\beta }_{sa,z}\right)+\left({PCA}_{ls}*{\beta }_{ls,z}\right))/5$$

where *CompWght*_*z*_ represents the *z*th component weight of *n* that are possible, *PCA*_*j*_ represents the factor loading from the first PCA component for subjective outcome *j*, and $\beta$_j,z_ represents the coefficient for component *z* from the regression on subjective outcome *j*.

After calculating Eq. 1) for each of the components to arrive at their respective index weight, these values can in turn be used to score well-being for individual respondents. This is done by multiplying the individual response for each component (zero or one) by the corresponding component weight, and then summing over all of the components. We call this measure the Child and Adolescent Thriving Index 1.0. The final index score for respondent *i* (*CATI*_*i*_) is presented in Eq. 2) below:

- $${CATI}_{i}=\sum_{z=1}^{n}{CompWght}_{z}*{Comp}_{i,z}$$

where *Comp*_*z*_ represents the respondent’s value for the *z*th component of *n* that are possible, and *CompWght*_*z*_ represents the corresponding component weight take from Eq. 1) above.

As we have mentioned, the primary advantage of this *hedonic* weighting approach is that it places greater emphasis on the subjective perspective when deriving an index (Decancq & Lugo, 2013), thereby achieving our aim of making the measure more child-centric. However, there are several limitations to the approach. First, as already discussed, well-being is a multidimensional concept that cannot be represented by one subjective measure. We have tried to partially account for this by using multiple subjective outcomes, but important constructs will inevitability be omitted, and the usage of PCA to reduce data dimensionality further results in some loss of information within the scales that we have. Second, an additive index such as ours presents problems of substitutability, whereby deficiencies in certain aspects of well-being can be canceled out by improvements in others (Biggeri & Ferrone, 2021). This is a somewhat inevitable tradeoff when simplifying the measurement to a single arithmetic formula. Third, despite the appearance of a fully-automated and rigorous mathematical procedure, there are certain subjective decisions that need to be made by the researcher, such as whether to include or drop components when the relationship with well-being is of moderate strength and how to accommodate for situations where components are highly correlated or even multicollinear with one another. The aforementioned sensitivity analysis (Appendix Table 10) shows these decisions do not greatly influence the broad findings, but the subjectivity of the overall methodology should still not be completely overlooked.

### Predictive Validity of the Index

To assess whether our methodology results in a useful metric for population health assessment, we adopt a predictive validity test using outcomes collected in young adulthood. In each wave of the TAS, respondents are asked about their general health (excellent/very good/good/fair/poor) and whether they were depressed for at least 2 weeks in the past year. Both of these measures have been shown to be associated with future health outcomes such as mortality and morbidity (Benjamins et al., 2004; Burström & Fredlund, 2001; Christensen et al., 2017; Cullati et al., 2018; St John & Montgomery, 2009). We construct dichotomous measures for whether the respondent ever indicated fair/poor health during young adulthood and whether the respondent ever indicated depression during young adulthood. Each measure was then regressed against the demographic controls as well as their standardized well-being index score. We use predictive margins to estimate the percentage increase in likelihood of reporting each young adulthood outcome based on a one standard deviation increase in index score. We present these marginal effects and their 95% confidence intervals, as well as the partial R^2^ of the model measure relative to one that only controls for demographic measures.

We perform an additional predictive validity analysis on a constructed measure of peak individual earnings between ages 20 and 29, which is drawn from questions in the main PSID file. The measure is available for 98% of the sample (see Appendix Fig. 3 for the distribution). We follow a similar methodology to the one described above, but use poisson regression with robust standard errors and control for demographics, number of observed years aged 20–29, whether enrolled during year of highest earnings, and household position during year of highest earnings (head [reference]; spouse; neither).

We then conduct several subanalyses using the results of the predictive validity analysis to address the secondary aims of this paper. First, the results are compared to an index constructed using a set of individual-level measures similar to those of a widely-adopted index, the Annie E. Casey’s KIDS COUNT index (The Annie E. Casey Foundation, 2021). Components used in this recreation of KIDS COUNT are shown in Appendix Table 11. The index score is constructed by counting the number of positive outcomes each child has (which has the effect of equally weighting the components) and then standardizing the measure. Second, we compare the predictive validity results of our methodology using only individual-level components with our methodology using individual-level and contextual components. Lastly, we construct versions of our index using the self-reported health and earnings measures from young adulthood (general health, depression, and peak earnings) as the benchmark measures for deriving weights, rather than the five subjective scales in our main specification. For all three subanalyses, we perform seemingly unrelated estimation analysis to assess whether estimates are significantly different from the main index specification.

## Results

### Analytic Sample

Table 1 shows descriptive statistics for the analysis sample. The sample appears more at-risk than the general population, although the longer time frames for many of the component outcomes complicate their comparability with population-level cross sectional data. Comparing the unweighted and weighted estimates suggests any effect of attrition on the results are likely to be minor. Nonetheless we use attrition weights in later analysis.

**Table 1:** Descriptive Statistics of PSID Sample

Measures	Mean Including Imputed Observations; N = 2,942		Observed Sample Statistics (unweighted)		
	Unweighted	Weighted	Mean	σ	N
Subjective Well-Being in Young Adulthood (Age 18–28)					
Flourishing	13.65	13.62	13.65	2.06	2940
Psychological Distress	5.04	4.98	5.04	3.15	2941
Economic Worry	3.51	3.54	3.51	1.35	2893
Social Anxiety	3.36	3.42	3.36	1.22	2942
Life Satisfaction	2.26	2.22	2.25	0.67	2873
Candidate Components from Childhood – Individual					
Ever Food Insecure	13.3%	11.7%	13.3%	-	2934
Never in Preschool	62.4%	57.9%	62.6%	-	2925
Didn’t Graduate High School on Time	20.0%	16.9%	20.2%	-	2727
Ever Nonproficient in Math	75.6%	67.6%	75.9%	-	2759
Ever Nonproficient in Reading	73.5%	67.2%	73.6%	-	2751
Low Birthweight	27.1%	25.1%	27.0%	-	2910
Ever Obese	32.7%	30.2%	32.8%	-	2894
Ever in Fair/Poor Health	6.1%	5.6%	6.1%	-	2935
Ever Smoked Regularly	25.8%	27.0%	25.8%	-	2942
Ever Drank Regularly	17.7%	18.3%	17.2%	-	2471
Tried Marijuana	49.9%	49.1%	49.9%	-	2941
Ever Pregnant	24.7%	17.7%	24.7%	-	2942
Ever Arrested	16.5%	13.6%	16.5%	-	2942
Candidate Components from Childhood – Contextual					
Ever in a Housing Burdened Household (Costs > 1/3 income)	60.5%	55.7%	48.9%	-	2942
Ever in Household Poverty	46.2%	38.4%	49.9%	-	2942
Ever in a Household with Parent Unemployed	51.7%	44.4%	50.0%	-	2942
Family Ever Moved	81.7%	78.0%	38.7%	-	2942
Ever in a Household Where No Parent Had High School Degree	26.5%	23.7%	44.1%	-	2942
Ever in Household With Unmarried Head	73.8%	68.7%	44.0%	-	2942
Predictive Validity Outcomes from Adolescence					
Ever in Fair/Poor Health	21.2%	20.2%	21.2%	-	2942
Ever Depressed 2 Weeks in Past Year	29.3%	28.3%	29.3%	-	2942
Highest Earnings Between Ages 20–29 (2018 dollars)	$31,259.38	$33,065.23	$31,259.38	$27,664.18	2889

PSID = Panel Study of Income Dynamics. σ indicates standard deviation. “- “ indicates standard deviation not shown since variable is dichotomous.

### Primary Aims—Developing the Index and Assessing Predictive Validity

Table 2 shows principal component analysis results for the standardized average subjective outcomes during young adulthood. The eigenvalue of the first principal component, which indicates how much of the variability across the five subjective outcomes is explained, is 2.62. This is the only eigenvalue that exceeds the standard threshold of 1, indicating that the decision to use the factor loadings from only the first principal component is reasonable. Furthermore, the first principal component’s corresponding factor loading for each subjective outcome has the anticipated direction we would expect from a measure of well-being: psychological distress, economic worry, and social anxiety all have negative signs, while flourishing and life satisfaction have positive signs. The magnitude of the coefficients for all five subjective outcomes are similar, with a slightly greater size for psychological distress (-0.502), economic worry, (-0.475), and flourishing (0.475) than for social anxiety (-0.392) and life satisfaction (0.378).

**Table 2:** Principal Component Analysis of PSID-TAS Subjective Scales

Component	Eigenvalue				
Component 1	2.62				
Component 2	0.92				
Component 3	0.55				
Component 4	0.48				
Component 5	0.42				
Subjective Scale	Factor Loading				
	1	2	3	4	5
Flourishing	0.475	-0.245	-0.608	-0.234	-0.538
Kessler’s K-6 Non-Specific Psychiatric Distress	-0.502	-0.110	0.060	0.512	-0.685
Economic Worry	-0.475	-0.149	-0.742	0.154	0.421
Social Anxiety	-0.392	-0.648	0.229	-0.610	-0.045
Life Satisfaction	0.378	-0.697	0.151	0.536	0.249

N = 2,942. PSID = Panel Study of Income Dynamics. TAS = Transition to Adulthood Supplement. Factor loadings for the first component are used as weights to aggregate subsequent regression results into final index.

Table 3 shows the regression results for each of the subjective outcomes after the variable selection process. Across the five subjective outcomes, the covariates that are always included are on-time high school graduation and food secure. The model selection procedure appears to work well at removing less important covariates: despite a somewhat generous inclusion criteria of p-value < 0.25 to be included in the final model, the vast majority of coefficients are significant at the 0.10 level or lower. On-time high school graduation is notable in that it is frequently significant at the 0.05 level. Not smoking regularly in adolescence is usually included as well, and typically has p-values significant at the 0.05 level.

**Table 3:** Model Selection and Final Regression of Subjective Outcomes on Candidate Index Components

Variable	Flourishing	Psychological Distress	Economic Worry	Social Anxiety	Life Satisfaction
Economic					
Food Secure	0.168* (0.098)	-0.166* (0.084)	-0.228** (0.087)	-0.177** (0.082)	0.171* (0.094)
Education					
Attended Preschool	POST	-0.102** (0.045)	POST	LASSO	LASSO
Graduated High School On Time	0.274*** (0.074)	-0.224** (0.086)	-0.357*** (0.080)	-0.171** (0.079)	0.273*** (0.063)
Math Proficiency	POST	LASSO	-0.121** (0.058)	-0.118* (0.067)	LASSO
Reading Proficiency	0.163*** (0.050)	-0.067 (0.046)	POST	POST	LASSO
Health					
Non-Low Birthweight	0.123*** (0.055)	LASSO	-0.096* (0.053)	LASSO	LASSO
Non-Obese	LASSO	POST	-0.084* (0.046)	LASSO	LASSO
Not in Fair/Poor General Health	LASSO	POST	LASSO	-0.136 (0.110)	LASSO
Health Behaviors					
Didn’t Smoke Regularly in Adolescence	0.236*** (0.069)	-0.224*** (0.065)	-0.267*** (0.055)	POST	0.104* (0.053)
Didn’t Try Marijuana in Adolescence	POST	-0.130** (0.061)	POST	POST	0.192*** (0.047)
Never Pregnant	LASSO	LASSO	LASSO	POST	LASSO
Family, Peers, Community					
Never Arrested	POST	LASSO	LASSO	POST	0.141* (0.079)
R^2^	0.057	0.049	0.075	0.039	0.082

Standard errors shown in parentheses. * (**)[***] = p-value is less than 0.10 (0.05) [0.01]. Models also control for average age during TAS responses, sex, and race/ethnicity. See Supplementary Material—Model Selection Methodology for more detail on the model-selection process. LASSO = Covariate Removed by Adaptive Least Absolute Shrinkage and Selection Operator procedure. POST = Covariate removed post-LASSO (*p*-value > 0.25 and/or sign is in opposite direction that is intended). Results for the index using individual and contextual components can be found in post-LASSO column of Appendix Table 10.

It should be noted that the R^2^ values for each of the regression equations is quite low (ranging from 0.039 to 0.082). While there is a case to be made that a very high R^2^ value is undesirable, since it would suggest well-being could be strongly predicted by indicators which are themselves unequally distributed within society, the values here suggest that there is substantial room for improvement in identifying outcome indicators that adequately proxy the well-being of children and adolescents.

Table 4 presents the final weights of the Child and Adolescent Thriving Index 1.0, which are the combined results of Tables 2 and 3 as described in the methods. Results are subsequently normalized so that each column sums to 1, for interpretability. The components with the largest weights are graduating high school on time (0.285), food secure (0.199), and not smoking regularly in adolescence (0.192), indicating these components are important signifiers of individual-level well-being. Taken together, they account for over two-thirds of the weight values (67.6%), despite comprising less than one-third of the included components (27.3%). The weights of the index are fairly robust to several alternative adjustments to the model selection procedure, indicating the choice of main specification does not heavily bias the results (Appendix Table 12).

**Table 4:** Final Weights For Child and Adolescent Thriving Index 1.0

Variable	Index Weight
Economic	
Food Secure	0.199
Education	
Attended Preschool	0.025
Graduated High School on Time	0.285
Math Proficiency	0.052
Reading Proficiency	0.054
Health	
Non-Low Birthweight	0.052
Non-Obese	0.020
Not in Fair/Poor General Health	0.027
**Health Behaviors**	
Didn’t Smoke Regularly in Adolescence	0.192
Didn’t Try Marijuana in Adolescence	0.069
Family, Peers, Community	
Never Arrested	0.027

Index weights are constructed by the following procedure: 1) multiply the absolute value of the product of the regression estimates for each subjective outcome presented in Table 3 with its respective PCA coefficient from Component 1 in Table 2; 2) averaging across the 5 outcomes; and 3) standardizing such that the column sums to 1. Results for the index using individual and contextual components can be found in Appendix Table 12.

Table 5 shows the results of the predictive validity exercise. Overall, higher scores on the Child and Adolescent Thriving Index 1.0 are related to improved outcomes in young adulthood at the population-level. A one-standard-deviation improvement in the index, controlling for demographic factors, is associated with a 7.9 percentage point decrease [95% CI: 5.9 – 9.8] in ever reporting fair or poor health during young adulthood, a 6.3 percentage point decrease [95% CI: 4.6 – 8.0] in ever reporting depressive symptoms during young adulthood, and a 17.2 percent increase [95% CI: 13.7 – 20.5] in highest earnings between ages 20–29. These results are not strongly affected by the model selection procedure, suggesting our choice of preferred approach does not heavily influence the results (Appendix Table 13).

**Table 5:** Predictive Validity of Child and Adolescent Thriving Index 1.0 on Young Adulthood Outcomes

Outcome in Young Adulthood	Ever in Fair / Poor Health (percentage point change)		Ever Depressed 2 Weeks in Past Year (percentage point change)		Highest Earnings Between Ages 20–29 (percent change)	
Statistic	Marginal Effect	Partial R^2^	Marginal Effect	Partial R^2^	Marginal Effect	Partial R^2^
Preferred Specification						
Child and Adolescent Thriving Index 1.0 (I)	-0.079 (-0.059 – -0.098)	0.029 (0.045 vs. 0.016)	-0.063 (-0.046 – -0.080)	0.019 (0.033 vs. 0.014)	0.172 (0.137 – 0.205)	0.042 (0.312 vs. 0.270)
Subanalysis i) *Comparing to existing methodology*						
KIDS COUNT	-0.075 (-0.055 – -0.096)	0.026 (0.042 vs. 0.016)	-0.057 (-0.039 – -0.075)	0.015 (0.029 vs. 0.014)	0.176 (0.143 – 0.208)	0.044 (0.314 vs. 0.270)
Subanalysis ii) *Comparing to methodology using contextual components*						
Child and Adolescent Thriving Index 1.0 (I & C)	-0.084 ** (-0.065 – -0.103)	0.035 (0.051 vs. 0.016)	-0.068 (-0.050 – -0.085)	0.023 (0.037 vs. 0.014)	0.179 (0.147 – 0.210)	0.047 (0.317 vs. 0.270)
Subanalysis iii) *Comparing to methodology using self-reported health or earnings as benchmarks*						
Health Fair/Poor (I)	NA	NA	-0.051 (-0.031 – -0.071)	0.012 (0.026 vs. 0.014)	0.137 *** (0.104 – 0.169)	0.030 (0.300 vs. 0.270)
Depressed (I)	-0.071 ** (-0.050 – -0.090)	0.020 (0.036 vs. 0.016)	NA	NA	0.125 *** (0.088 – 0.160)	0.020 (0.290 vs. 0.270)
High Income (I)	-0.063 ** (-0.043 – -0.083)	0.017 (0.033 vs. 0.016)	-0.051 * (-0.034 – -0.068)	0.012 (0.026 vs. 0.014)	NA	NA

Marginal effect estimates show effect of a one standard deviation increase of well-being score on later health, with 95% Confidence Interval in parentheses. Marginal effect estimates use *mimrgns* user-written command in STATA. Underlying regression models control for health outcomes control for age, sex, and race/ethnicity. For highest earnings column, regression models also control for observed years between ages 20–29, whether enrolled at time of highest earnings, and household position at time of highest earnings (head [reference], spouse, other).

(I) indicates index is restricted to individual-level components only. (I & C) indicates index allows for both individual-level and contextual components.

*(**)[***] indicates estimate is significantly different from Child and Adolescent Thriving Index 1.0 (I) at 0.10 (0.05) [0.01] level using seemingly unrelated estimation.

Partial R^2^ shows the increase in R^2^ when adding the index score to a model controlling for demographic factors, with the respective R^2^ for each model shown in parentheses.

### Secondary Aims – Comparing Predictive Validity with Alternative Methodologies

The bottom 3 panels of Table 5 show the results for the several secondary analyses. First, the predictive validity of the Child and Adolescent Thriving Index 1.0 is not significantly different than that of the reconstructed KIDS COUNT index. A one-standard-deviation increase in KIDS COUNT is associated with the following outcomes in young adulthood: a 7.5 percentage point decrease [95% CI: 5.5 – 9.6] in ever reporting fair or poor health, a 5.7 percentage point decrease [95% CI: 3.9 – 7.5] in ever reporting depressive symptoms, and a 17.6 percent increase [95% CI: 14.3 – 21.0] in highest earnings between ages 20–29. None of these estimates are statistically significantly different at the 0.05 level from the corresponding estimates using our methodology above.

Incorporating additional information into the index about the surrounding context leads to a slight improvement in predictive validity. A one-standard-deviation increase in the index using both individual and contextual-level information is associated with the following outcomes in young adulthood: a 8.4 percentage point decrease [95% CI: 6.5 – 10.3] in ever reporting fair or poor health, a 6.8 percentage point decrease [95% CI: 5.0 – 8.5] in ever reporting depressive symptoms, and a 17.9 percent increase [95% CI: 14.7 – 21.0] in highest earnings between ages 20–29. The only outcome for which predictive validity is significantly better using the contextual index is ever in ever reported fair/poor health (p = 0.022).

Adopting measures of self-reported health or earnings in young adulthood as benchmarks for deriving index weights, rather than the five subjective scales, leads to worse performance in predicting future outcomes. The marginal effects of a one standard deviation increase in index score on future outcomes are smaller when using these alternatives, with four of the six seemingly unrelated estimations significant at the 0.05 level. We omit predictions where the same measure would be used as both the benchmark and the predicted outcome.

Regardless of the specification, the indices improve the ability to predict worse outcomes in young adulthood over demographic information alone. However, even the models with the best predictive ability—those that incorporate all of the individual and contextual information available on the PSID—have a partial R^2^ value of only 0.035 for fair/poor health status, 0.023 for depression in the past year, and 0.047 for highest earnings between ages 20–29, suggesting that the well-being index does not perform well as a diagnostic tool at the individual level.

## Discussion

This analysis uses data from a longitudinal panel to devise an index of the outcome indicators of children and adolescent well-being in the United States. The methodology presented here represents several advances in theory and analytic techniques, all while remaining compatible with existing population data infrastructure. Our principal findings are that first, the latent construct of child and adolescent well-being can be usefully tracked at the population-level with a relatively small set of outcomes that are already collected at scale, with the three standing out as most important being on-time high school graduation, food security, and not smoking regularly during adolescence. Second, the index displays strong predictive validity for several important young-adulthood outcomes, supporting its use as a population-level monitoring tool. The predictive validity subanalyses unearth several secondary findings: i) the preeminent measure of child well-being, the KIDS COUNT index, performs similarly to our index; ii) incorporating contextual-level measures does not substantially improve the index; and iii) using subjective scales to derive weights, rather than self-reported health or earnings, is preferable.

The resulting index makes several methodological improvements to previous alternatives. This is accomplished by allowing for index components to take on different levels of importance, through two methodological innovations. First, model selection techniques remove less important components. Then, subjective outcomes are regressed on the remaining components. These methods attenuate biases that could be introduced by relying on expert opinion, a valid concern given previous work has demonstrated that the priorities of the researcher and their subject may not be well-aligned (Raphael et al., 1996). Even in cases where expert opinion is informed by the experiences of children, other forms of bias, such as double-counting certain aspects of well-being at the expense of others, necessitate a more data-driven method like the one implemented here.

The index shows good utility as a population health measure: an increased score is associated with lower likelihoods of worse physical and mental health in young adulthood, after controlling for demographic factors. However, the index does not demonstrate strong capability as a diagnostic tool – with a partial adjusted R^2^ less than 0.05 in these models, its ability to predict well-being at the individual level is limited. There is precedent for instruments that do not perform well at the individual level still holding great value as a population health measure, such as body mass index (Gutin, 2018). Nevertheless, reliably identifying individual children and adolescents with poor well-being using only measures commonly available at the population-level does not appear feasible at the present time.

The index makes several conceptual improvements to existing measurement efforts, and then assesses these contributions empirically. First, it removes components that are contextual determinants of well-being, such as household poverty and housing burden. When comparing its predictive capabilities with those of an index that does allow for these contextual determinants, the results are essentially identical. This is important if these indices are to be used as an evaluative outcome in the future. Public policy has the capacity to promote well-being by shaping the contexts in which children and adolescents live, whether it be their family, school, or community. By carefully separating these contexts from the final outcome measure, the index exhibits stronger face validity as a tool for scientific inquiry and evaluation.

Second, this index incorporates the subjective perspective for the first time in the American context. This is important because the objective and subjective well-being viewpoints, each with their own rich tradition of scholarship and inquiry, are complements to one another. This is most clearly evidenced empirically in several studies that have found a weak direct relationship between the two (Kahneman & Krueger, 2006). In our analysis, we find that an index using a set of subjective scales to derive component weights outperforms a comparable set of indices relying on relatively more objective measures such as health or earnings. This should provide further support for existing defenses of subjective measures against those who view them unreliable (Bradshaw, 2019; Cummins & Land, 2018).

Despite these empirical and conceptual improvements, the difference between the main index specifications and a reconstructed version of the KIDS COUNT index in predicting future health issues is slight. Most likely, this is the result of a limited set of candidate index components available at the population-level. This is evidenced by the low R^2^ values in the main regression tables, which indicate that much of the variance in the subjective outcomes remains unexplained by the sort of data which is readily collected at the population level. In the long term, data systems at both the national and state-level should be further expanded to track a broader range of objective and subjective measures corresponding to well-being, so that future indices have a deeper pool of potential components to draw from. In the interim, advocates and policy makers already relying on KIDS COUNT can continue to use the index (including historical estimates) as a reasonably adequate proxy of well-being, with the knowledge that these indices perform well against more rigorous methodologies.

There are several limitations worth noting. First, our subjective outcomes for benchmarking the index are collected during young adulthood, rather than during childhood or adolescence as one might expect for an index aimed at this stage of the lifecourse. We do this for two reasons: 1) this allows for the entire set of index components to fully occur (i.e. high school graduation, substance use in adolescence, etc.); and 2) the PSID collects a larger number of subjective scales in young adulthood. We perform a robustness test by recreating the procedure using two subjective scales collected during childhood and adolescence as the basis for deriving weights. Appendix Table 14 shows that the results are fairly similar to using the corresponding subjective measures in young adulthood.

The benefits of relying on the subjective perspective for benchmarking the index have been well-described, but there are several potential weaknesses of the approach. Subjective measures have been shown to be influenced by questionnaire design, such as the ordering of responses and scaling effects (Podsakoff et al., 2003). Furthermore, there is some measurement error inherent to relying on a single point in time instrument, since it is quite likely that levels of stress, mood, and other psychological factors may influence the respondent. Lastly, subjective measures are seen as less reliable by many, in part because they are frequently uncorrelated, or in some instances negatively correlated, with measures that are objectively beneficial to individuals, as seen with teen pregnancy in this analysis (Hardoon et al., 2003). However, even in the presence of these limitations, subjective measures may still outperform more objective instruments in certain situations, as seen in our predictive validity analysis (Jahedi & Méndez, 2014), and concerns typically raised related to relying on a young person’s perspective are often overstated (Bradshaw, 2019).

Other limitations include an absence of certain measures that should correspond with higher levels of well-being among children, such as relationship quality with family and friends, connection to school and other institutions, and hope for the future (Chi et al., 2019; Cho, 2018; Hagen et al., 2005; Kühner et al., 2021; Monahan et al., 2010; Snyder, 2005; Thompson et al., 2006). These data gaps are driven by two forms of availability our index must consider: availability on the PSID and availability at the population-level for subsequent analysis. Furthermore, the cohort of children in this study represent a particular time and period in the United States context that may not translate well to measuring well-being in other settings. Widespread adoption of smartphone technology and social media, changing patterns in youth smoking, the societal fallout from the COVID-19 pandemic, and rising levels of anxiety related to emerging threats such as climate change or the failure of political and social institutions are just a few examples of how influences on well-being may have changed between this cohort and the next generation of children. Additionally, the design of the PSID does not facilitate more detailed, age-specific collection of component variables. This is an important limitation to note, given the extensive body of research detailing that adversities experienced earlier in childhood tend are more likely to have large, lifelong effects (Duncan et al., 1998; Gariepy et al., 2017; Halfon et al., 2014; Morrissey & Kinderman, 2020). Furthermore, because the model is focused on measurement rather than causal frameworks, components occurring in later childhood and adolescence may be more likely to have higher weights, since they occur closer in time to when subjective measures are collected. Lastly, some vulnerable populations like foster youth and children experiencing homelessness are likely underrepresented in our study population.

These limitations suggest that the Child and Adolescent Thriving Index 1.0, as derived here, is likely not the optimal composite measure of well-being. Nonetheless, this paper provides a proof of concept for a methodology that could derive a stronger measure of well-being when used with a longitudinal data source specifically designed for this task.

## Conclusion

Population health assessment and monitoring is an essential function of public health. To fulfill this responsibility, well-being has been highlighted by some as a key concept for understanding the nation’s health, and yet it does not have an agreed upon system of measurement. This analysis implements a new methodology for an index that aims to summarize the outcome indicators of well-being during childhood and adolescence. Our measure makes several theoretical and methodological improvements to prior indices – more fully incorporating the subjective perspective, restricting components to those assessed at the individual-level, and allowing components to be weighted differently – each of which makes it more child-centric. We find that the outcome indicators of child and adolescent well-being can be reasonably assessed at the population-level using several commonly available measures, and that this index is associated with improved health and earnings in young adulthood.

A common adage in public policy is “what gets measured gets improved.” While it is broadly understood that well-being is important, a rigorous system of measurement is needed to make crucial advances in this area of population health. Therefore we recommend several promising areas of future work that can build upon our findings. First, researchers should seek to build new and improved longitudinal cohorts more specifically designed for this task, and assess whether such efforts can boost predictive power of the index and address other limitations in this work. Second, future work should explore developing indices for specific subpopulations, since the outcome indicators of well-being may vary in their strength of association with subjective scales across gender, race/ethnicity, and socioeconomic position. Third, researchers should implement this index methodology using population-level macrodata to see if it results in different conclusions for national and state-level trends. Lastly, as these indices continue to improve, they should begin to serve as outcome measures for assessing the impacts of various policies.

## Supplementary Information

Below is the link to the electronic supplementary material.

Supplementary file1 (DOCX 426 KB)
