# Supplementary material for: The Child and Adolescent Thriving Index 1.0: Developing a Measure of the Outcome Indicators of Well-Being for Population Health Assessment
Source: Child Indic Res. 2022 Aug 6;15(6):2015–42. doi: 10.1007/s12187-022-09962-0 (PMC9362709; doi:10.1007/s12187-022-09962-0)
Supplement: Supplementary file 1 — Supplementary file1 (DOCX 426 KB) [file 12187_2022_9962_MOESM1_ESM.docx]

**Appendix Material**

*Page Number*

Figure 2. Sample Layout 2

Table 6. PSID Subjective Scale Components 3

Table 7. Well-Being Candidate Components 5

Table 8. Multiple Imputation Descriptives 8

Table 9. Post-LASSO Model Step-by-Step Selection Process 9

Table 10. Pre and Post-LASSO Model Comparison 11

Figure 3. Distribution of Highest Earnings During Ages 20-29 13

Table 11. Annie E. Casey KIDS COUNT Index and Data Sources 14

Table 12. Final Weights For Alternate Specifications of Index 15

Table 13. Marginal Effect and Partial R2 of Increased Index Score on Young Adulthood Outcomes 16

Table 14. Comparison when Using Subjective Measures from Childhood and Adolescence 17

**Figure 2**

*Sample Progression Through the PSID*


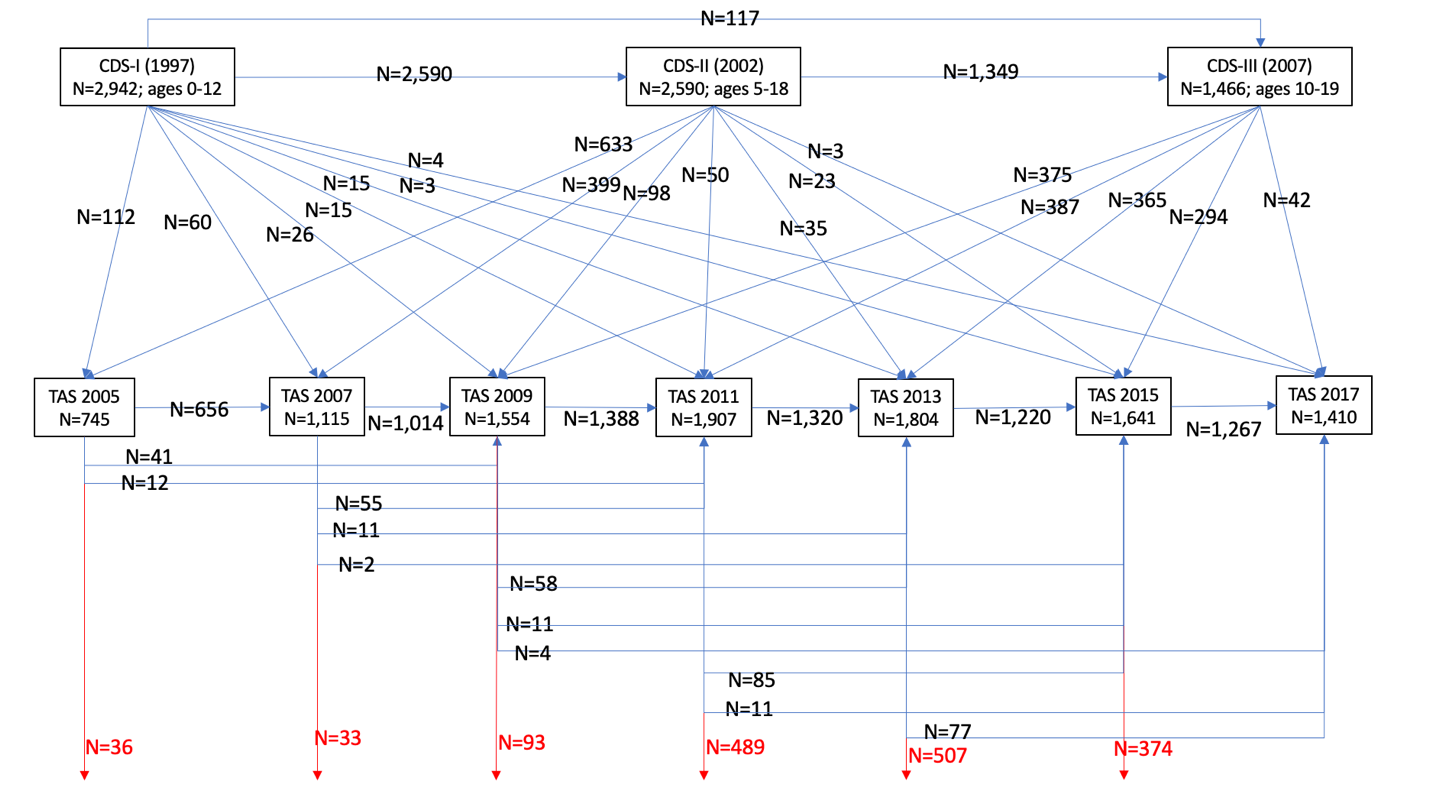


Notes: PSID = Panel Study of Income Dynamics. CDS = Child Development Supplement. TAS = Transition to Adulthood Supplement. Skip patterns are denoted by arrows, with each one having a marker that shows how many observations followed the pattern. Red arrows and numbers indicate when respondents left the sample prior to TAS 2017.

**Table 6**

*PSID Subjective Scale Components*

| **Subjective Scale** | **Form** | **Components** |
| --- | --- | --- |
| Flourishing  (TAS 2005, 2007, 2009, 2011, 2013, 2015, and 2017) | For each question, the respondent can answer one the following: never, once or twice, about once a week, two or three times a week, almost every day, and every day.  Each item is assigned a score ranging from 0-2 based on this response | 1. In the last month, how often did you feel happy? 2. In the last month, how often did you feel interested in life? 3. In the last month, how often did you feel satisfied? 4. In the last month, how often did you feel that you had something important to contribute to society? 5. In the last month, how often did you feel that you belonged to a community (like a social group, your school, or your neighborhood)? 6. In the last month, how often did you feel that our society is becoming a better place? 7. In the last month, how often did you feel that people are basically good? 8. In the last month, how often did you feel that the way society works made sense to you? 9. In the last month, how often did you feel good at managing the responsibilities of your daily life? 10. In the last month, how often did you feel that you have warm and trusting relationships with other kids? 11. In the last month, how often did you feel that you have experiences that challenged you to grow or become a better person? 12. In the last month, how often did you feel confident to think or express your own opinions? 13. In the last month, how often did you feel that you liked your personality? 14. In the last month, how often did you feel that your life had a direction or purpose? |
| Psychological Distress  (TAS 2005, 2007, 2009, 2011, 2013, 2015, and 2017) | The scale is constructed from a series of 6 questions which assesses their experiences in the past month(Kessler et al., 2003). For each question, the respondent is assigned a score ranging from 0-4, where 0 indicates the absence of a negative outcome and 4 indicates the strong presence of a negative outcome. | 1. During the past 30 days, how often did you feel nervous? 2. During the past 30 days, how often did you feel hopeless? 3. During the past 30 days, how often did you feel restless or fidgety? 4. During the past 30 days, how often did you feel that everything was an effort? 5. During the past 30 days, how often did you feel so sad that nothing could cheer you up? 6. During the past 30 days, how often did you feel worthless? |
| Economic Worry  (TAS 2005, 2007, 2009, 2011, 2013, and 2015) | Respondents are asked to assign the following items a score from 1 to 7, where 1 indicates “Never” and 7 indicates “Daily.” | 1. How often do you worry that you may not have enough money to pay for things? 2. How often do you worry that you will not have a good job in the future? 3. How often do you feel discouraged about the future? |
| Social Anxiety  (TAS 2005, 2007, 2009, 2011, 2013, 2015, and 2017) | Respondents are asked to assign the following items a score from 1 to 7, where 1 indicates “Never” and 7 indicates “Daily.” | 1. How often do you feel nervous when meeting new people? 2. How often do you feel shy? 3. How often do you feel self-conscious when you’re around people? 4. How often do you feel nervous about performing in front of others or making a presentation? |
| Life Satisfaction  (TAS 2009, 2011, 2013, 2015, and 2017) | Completely Satisfied, Very Satisfied, Somewhat Satisfied, Not Very Satisfied, Not at All Satisfied | Please think about your life as a whole. How satisfied are you with it? |

| **Table 7**  *Well-Being Candidate Components* | | |
| --- | --- | --- |
| Candidate Component | PSID Question (Supplement ; Year) |  |
| **Section 1) Individual-Level Components** |  |  |
| Economic |  |  |
| Food Insecurity^1^ | ER14331X (Family ; 1999)  ER18470X (Family ; 2001)  ER21735X (Family ; 2003)  ER60802 (Family ; 2015) |  |
|  |  |  |
| Education |  |  |
| Among age 3-4, not enrolled in school in the previous three months | ER33261 and ER33264 (Individual ; 1995)  Q1G2A (CDS ; 1997)  Q1H12F and Q1H12G (CDS ; 1997)  Q1H4_1 through Q1H4_7 (CDS ; 1997)  Q21B5 (CDS ; 2002)  Q21B15N (CDS ; 2002)  Q21C3_1 through Q21C3_4 (CDS ; 2002)  Q31B15N (CDS ; 2007) |  |
|  |  |  |
| 8th grade public school students not proficient in reading | Q3BRPR (CDS ; 1997)  Q24BRPR (CDS ; 2002)  Q34BRPR (CDS ; 2007) |  |
|  |  |  |
| 8th grade public school students not proficient in math | Q3BMPR (CDS ; 1997)  Q24APPR (CDS ; 2002)  Q34APPR (CDS ; 2007) |  |
|  |  |  |
| Entering freshman class not graduating on time / dropping out | Q21B36 and Q21B36A (CDS ; 2002)  Q21B27 and Q21B27I through Q21B2L (CDS ; 2002)  Q31B36 and Q31B36A (CDS ; 2007)  Q31B27 and Q31B27I through Q31B2L (CDS ; 2007)  TA050573 (TAS ; 2005)  TA070548 (TAS ; 2007)  TA090590 (TAS ; 2009)  TA110671 (TAS ; 2011)  TA130691 (TAS ; 2013)  TA150701 (TAS ; 2015)  TA170781 (TAS ; 2017) |  |
|  |  |  |
| Physical Health |  |  |
| Percent of babies born with low birthweight (less than 5.5 pounds) | ER32014 (Individual ; all years)  Q1A8P and Q1A8O (CDS ; 1997)  Q31A0_LB and Q31A0_OZ (CDS ; 2007) |  |
|  |  |  |
| Rates of obesity | WTIND97 (CDS ; 1997)  WTIND02 (CDS ; 2002)  WTIND07 (CDS ; 2007) |  |
|  |  |  |
| Parent-reported general health | Q1G33A and Q5A10A (CDS ; 1997)  Q21A11 (CDS ; 2002)  Q31A11 (CDS ; 2007) |  |
| Family, Peers, and Community |  |  |
| Teen Pregnancy | Q23L33 (CDS ; 2002)  Q33L33 (CDS ; 2007)  TA050087, TA050090, and TA050092 (TAS ; 2005)  TA070087, TA070090, and TA070092 (TAS ; 2007)  TA090096, TA090099, and TA090101 (TAS ; 2009)  TA110097, TA110100, and TA110102 (TAS ; 2011)  TA130096, TA130099, and TA130101 (TAS ; 2013)  TA150088, TA150091, and TA150093 (TAS ; 2015)  TA170172 and TA170174 (TAS ; 2017) |  |
|  |  |  |
| Juvenile arrests | Q23L11J (CDS ; 2002)  Q33L11J (CDS ; 2007)  TA050860, TA050861, and TA050862 (TAS ; 2005)  TA070841, TA070842, and TA070843 (TAS ; 2007)  TA090901, TA090902, and TA090903 (TAS ; 2009)  TA111032, TA111033, and TA111034 (TAS ; 2011)  TA131067, TA131068, and TA131069 (TAS ; 2013)  TA151107, TA151108, and TA151109 (TAS ; 2015)  TA171937, TA171938, and TA171939 (TAS ; 2017) |  |
|  |  |  |
| Health Behaviors |  |  |
|  |  |  |
| Smoked Regularly | Q23L12B (CDS ; 2002)  Q33L12B (CDS ; 2007)  TA050761 and TA050765 (TAS ; 2005)  TA070732 and TA070736 (TAS ; 2007)  TA090791 and TA090795 (TAS ; 2009)  TA110907 and TA110911 (TAS ; 2011)  TA130940 and TA130944 (TAS ; 2013)  TA150956 and TA150960 (TAS ; 2015)  TA171810 and TA171814 (TAS ; 2017) |  |
|  |  |  |
| Used Alcohol Regularly | Q23L13 and Q23L13C (CDS ; 2002)  Q33L13 and Q33L13C (CDS ; 2007) |  |
|  |  |  |
| Tried Marijuana | Q23L14A (CDS ; 2002)  Q33L14A (CDS ; 2007)  TA050786 and TA050787 (TAS ; 2005)  TA070757 and TA070758 (TAS ; 2007)  TA090816 and TA090817 (TAS ; 2009)  TA110932 and TA110933 (TAS ; 2011)  TA130965 and TA130966 (TAS ; 2013)  TA150987 and TA150988 (TAS ; 2015)  TA171828 and TA171829 (TAS ; 2017) |  |
|  |  |  |
| **Section 2): Contextual-Level** |  |  |
| Economic |  |  |
| Ever Living in Household in Poverty | Income: V12371, V13623, V14670, V16144, V17533, V18875, V20175, V21481, V23322, ER4153, ER6993, ER9244, ER12079, ER16462, ER20456, ER24099, ER28037, ER41027, ER46935, ER52343, ER58152, ER65349, and ER71426 (Family ; 1984-1996 and every other year from 1998-2016)  Threshold: V12447, V13688, V14738, V16209, V17613, V18884, V20184, V21490, V23326, ER4155, ER6995, ER9246, ER12220, ER16427, ER20373, ER24140, ER28039, ER41029, ER46972, ER52396, ER58213, ER65449, and ER71528 (Family ; 1984-1996 and every other year from 1998-2016) |  |
|  |  |  |
| Ever have Unsecure Employment Situation (no parent employed) | Head: V11702, V13102, V14200, V15254, V16755, V18193, V19493, V20793, V22570, V11799, V13195, V14291, V15401, V16916, V18340, V19640, V20940, V22736, ER4101, ER6941, ER9192, ER12179, ER16476, ER20404, ER23702A2, ER24086, ER27711D3, ER27892, ER40686A2, ER40882, ER46666, ER46778, ER52067, ER52186, ER57797, ER57987, ER64977, ER65167, ER71069, and ER71244 (Family ; 1984-2016 except 1997 and 1999)  Spouse: V11251, V12650, V13802, V14858, V16358, V17767, V19067, V20367, V21664, V12065, V13279, V14373, V15556, V17074, V18495, V19795, V21095, V22923, V12162, V13363, V14455, V15703, V17235, V18642, V19942, V21242, V23089, ER2561A, ER5560A, ER7656A, ER10562A, ER13716, ER18926, ER22296, ER22296, ER25361, ER25361, ER36366, ER36366, ER42391, ER42391, ER47704, ER47704, ER53410, ER53410, ER60425, ER60425, ER66438, ER66438, ER4112, ER6952, ER9203, ER12190, ER16487, ER20415, ER23702F5, ER24097, ER27711F5, ER27903, ER40686F5, ER40893, ER46677, ER46799, ER52078, ER52207, ER57845, ER58008, ER65025, ER65188, ER71117, and ER71265 (Family ; 1984-2016 except 1997 and 1999) |  |
|  |  |  |
| Ever have High Housing Cost Burden | Housing: ER16515A5, ER20456A5, ER24138A5, ER28037A5, ER41027A5, ER46971A5, ER52395A5, ER58212A5, ER65414, and ER71491 (Family every other year from 1999-2017)  Income: ER16462, ER20456, ER24099, ER28037, ER41027, ER46935, ER52343, ER58152, ER65349, and ER71426 (Family every other year from 1998-2016) |  |
|  |  |  |
| Family, Peers, and Community |  |  |
| Household Head ever Unmarried | V10426, V11612, V13017, V14120, V15136, V16637, V18055, V19355, V20657, V22412, ER2014, ER5013, ER7013, ER10016, ER13021, ER17024, ER21023, ER25023, ER36023, ER33907, ER42023, ER47323, ER53023, and ER60024 (Family ; 1984-1997 and every other year from 1999-2017) |  |
|  |  |  |
| Ever live in a Household Where the Head or Spouse Lacks a HS Diploma or Equivalent | Head: V11042, V12400, V13640, V14687, V16161, V17545, V18898, V20198, V21504, V23333, ER4158, ER6998, ER9249, ER12222, ER16516, ER20457, ER24148, ER28047, ER41037, ER46981, ER52405, ER58223, ER65459, and ER71538 (Family ; 1984-1997 and every other year from 1999-2017)  Spouse: V11043, V12401, V13641, V14688, V16162, V17546, V18899, V20199, V21505, V23334, ER4159, ER6999, ER9250, ER12223, ER16517, ER20458, ER24149, ER28048, ER41038, ER46982, ER52406, ER58224, ER65460, and ER71539 (Family ; 1984-1997 and every other year from 1999-2017) |  |
|  |  |  |
| Ever Moved | V10447, V11628, V13037, V14140, V15148, V16649, V18087, V19387, V20687, V22441, ER2062, ER5061, ER7155, ER10072,, ER13077, ER17088, ER21117, ER25098, ER36103, ER42132, ER47440, ER53140, and ER60155 (Family ; 1984-1997 and every other year from 1999-2015) |  |
| Sources: PSID = Panel Survey of Income Dynamics ; Family = Main PSID family-level ; Individual = Main PSID individual-level ; CDS = Child Development Supplement ; TAS = Transition to Adulthood Supplement.  ^1^ Food insecurity uses variables for household food insecurity in instances where child status is missing. | | |
| Notes: Population data must be publicly available at the state or a smaller geography to be included in this table. | | |

**Table 8**

*Multiple Imputation Descriptives*

|  | Complete Cases | | | Remaining Sample | | | | Imputed Observations | | | | | | |
| --- | --- | --- | --- | --- | --- | --- | --- | --- | --- | --- | --- | --- | --- | --- |
|  | Mean | | SE | Mean | | | SE | Mean | | | | N | |  |
| **Outcomes** |  | |  |  | | |  |  | | | |  | |  |
| Flourishing | 13.71 | | 0.07 | 13.49 | | | 0.07 | 11.95 | | | | 2 | |  |
| K6 scale | 4.95 | | 0.09 | 5.04 | | | 0.11 | 8.07 | | | | 1 | |  |
| Econ. Worry | 3.53 | | 0.04 | 3.56 | | | 0.06 | 3.71 | | | | 49 | |  |
| Social Anxiety | 3.43 | | 0.04 | 3.42 | | | 0.05 | - | | | | - | |  |
| Life Satisfaction | 2.20 | | 0.02 | 2.25 | | | 0.02 | 2.33 | | | | 69 | |  |
|  |  | |  |  | | |  |  | | | |  | |  |
| **Individual Outcomes (Dichotomous)** |  | |  |  | | |  |  | | | |  | |  |
| Food insecurity | 12.1% | | - | 11.1% | | | - | . | | | | 8 | |  |
| Not in Pre-K | 56.6% | | - | 59.7% | | | - | 38.6% | | | | 17 | |  |
| Delay / Didn't Graduate HS | 13.8% | | - | 21.7% | | | - | 17.5% | | | | 215 | |  |
| Non-proficient in Math | 67.6% | | - | 67.5% | | | - | 66.9% | | | | 183 | |  |
| Non-proficient in Reading | 69.3% | | - | 64.0% | | | - | 69.9% | | | | 191 | |  |
| Low Birthweight | 23.5% | | - | 27.4% | | | - | 47.8% | | | | 32 | |  |
| Obese | 31.8% | | - | 27.6% | | | - | 30.2% | | | | 48 | |  |
| In Fair / Poor Health (parent-reported) | 6.2% | | - | 4.7% | | | - | - | | | | - | |  |
| Smoked Regularly in Childhood | 24.1% | | - | 31.3% | | | - | - | | | | - | |  |
| Drank Regularly in Childhood | 16.3% | | - | 21.3% | | | - | 21.4% | | | | 471 | |  |
| Tried Marijuana in Childhood | 46.9% | | - | 52.3% | | | - | - | | | | - | |  |
| Pregnant (or Impregnated) in Childhood | 17.5% | | - | 18.0% | | | - | - | | | | - | |  |
| Arrested in Childhood | 11.8% | | - | 16.4% | | | - | - | | | | - | |  |
|  |  | |  |  | | |  |  | | | |  | |  |
| **Contextual Outcomes (Dichotomous)** | |  |  | |  | | | | |  |  |  |  |  |
| Family Ever Housing Burdened (Costs >1/3 income) | 59.1% | | - | 50.5% | | | - | - | | | | - | |  |
| Family ever in Poverty | 36.7% | | - | 40.8% | | | - | - | | | | - | |  |
| Parent ever Unemployed | 44.5% | | - | 44.2% | | | - | - | | | | - | |  |
| Family Ever Moved | 78.8% | | - | 76.8% | | | - | - | | | | - | |  |
| Ever in Family with no Parent having HS Education or Equivalent | 21.8% | | - | 26.5% | | | - | - | | | | - | |  |
| Household Head Ever Unmarried | 72.7% | | - | 62.7% | | | - | - | | | | - | |  |
|  |  | |  |  | | |  |  | | | |  | |  |
| **Additional Variables for Multiple Imputation** | |  | |  | |  | | |  | | | |  |  |
| Chronic Absent (15+ days year during survey) | 6.0% | | - | 5.6% | | | - | - | | | | - | |  |
| Ever Expelled / Suspended | 16.6% | | - | 16.7% | | | - | 23.5% | | | | 40 | |  |
| Diagnosed With Asthma | 26.6% | | - | 22.6% | | | - | - | | | | - | |  |
| Doesn't Usually Eat Breakfast w/ Family | 30.5% | | - | 29.0% | | | - | - | | | | - | |  |
| Doesn't Usually Exercise 5+ days a week | 22.6% | | - | 21.4% | | | - | - | | | | - | |  |
| Doesn't Usually Sleep 8-12 Hours | 50.8% | | - | 47.7% | | | - | 47.9% | | | | 1109 | |  |
| Ever Hospitalized | 42.2% | | - | 46.6% | | | - | 42.1% | | | | 491 | |  |
| Childhood Flourishing | 13.08 | | 0.10 | 12.82 | | | 0.14 | 13.31 | | | | 893 | |  |
| Childhood K-6 Scale | 3.08 | | 0.10 | 3.42 | | | 0.17 | 2.76 | | | | 1330 | |  |
| Positive Behavior Scale | 4.17 | | 0.02 | 4.18 | | | 0.02 | 4.20 | | | | 13 | |  |
| Internalizing Scale | 3.38 | | 0.10 | 3.63 | | | 0.11 | 1.83 | | | | 361 | |  |
| Externalizing Scale | 5.70 | | 0.12 | 5.96 | | | 0.13 | 3.36 | | | | 91 | |  |
| Global Self-Concept Scale | 4.26 | | 0.02 | 4.55 | | | 0.03 | 4.34 | | | | 302 | |  |
| Parental Warmth Scale | 4.14 | | 0.02 | 4.16 | | | 0.03 | 3.90 | | | | 9 | |  |
| Parental Stress Scale | 2.18 | | 0.03 | 2.18 | | | 0.03 | 2.09 | | | | 158 | |  |
| Parental K-6 Scale | 4.52 | | 0.11 | 4.59 | | | 0.13 | 4.01 | | | | 399 | |  |
| Risky Scale | 1.38 | | 0.02 | 1.42 | | | 0.02 | - | | | | - | |  |
| Perceived Discrimination Scale | 2.50 | | 0.03 | 2.56 | | | 0.03 | - | | | | - | |  |
|  |  | |  |  | | |  |  | | | |  | |  |
| N | 1,760 | |  | 1,182 | | |  |  | | | |  | |  |

**Table 9**

*Post-LASSO Model Step-by-Step Selection Process*

| Subjective Outcome | Individual Model | | Individual & Contextual Model | |
| --- | --- | --- | --- | --- |
|  | Variable Removed | Reason | Variable Removed | Reason |
| Flourishing | Attended Preschool | p=.79 | Family Never Moved | p=.99 |
|  | Math Proficiency | p=.34 ; would be included when smoking removed instead | Attended Preschool | p=.96 |
|  | Didn’t Try Marijuana in Adolescence | p=.47 ; would be included when smoking removed instead |  |  |
|  | Never Arrested | p=.13 but wrong sign |  |  |
|  |  |  |  |  |
| Kessler K-6 | Non-Obese | p=.76 | Family Never Moved | p=.55 |
|  | Parent-reported Health | p=.56 | Parents Always Employed | p=.02 but wrong sign |
|  | Didn’t Drink Regularly in Adolescence | p=.12 but wrong sign ; became highly nonsignificant when smoking and marijuana removed instead |  |  |
|  |  |  |  |  |
| Economic Worry | Attended Preschool | p=.86 | Didn’t Try Marijuana in Adolescence | p=.66 ; would be included when smoking removed instead |
|  | Reading Proficiency | p=.68 ; would be included when math removed instead | Family Never Moved | p=.41 |
|  | Didn’t Try Marijuana in Adolescence | p=.37 ; would be included when smoking removed instead |  |  |
|  |  |  |  |  |
| Social Anxiety | Reading Proficiency | p=.29 ; would be included when math removed instead | Parent With HS Education or Equivalent | p=.90 |
|  | Never Pregnant | p=.28 ; wrong sign | Reading Proficiency | p=.33 ; would be included when math removed instead |
|  | Didn’t Try Marijuana in Adolescence | p=.01 but wrong sign ; would still be included with wrong sign if smoking removed instead | Never Pregnant | p=.26 ; wrong sign |
|  | Never Arrested | p=.01 but wrong sign ; would still be included with wrong sign if smoking removed instead | Didn’t Try Marijuana in Adolescence | p=.01 but wrong sign ; would still be included when smoking removed instead |
|  | Didn’t Smoke Regularly in Adolescence | p=.69 | Parents Always Employed | p=.01 but wrong sign |
|  |  |  | Never Arrested | p=.01 but wrong sign |
|  |  |  | Didn’t Smoke Regularly in Adolescence | p=.76 |
|  |  |  | Parent-Reported Health | p=.28 |
|  |  |  |  |  |
| Life Satisfaction | Didn’t Drink Regularly in Adolescence | p=.51 ; would be included if smoking and marijuana removed instead | Didn’t Drink Regularly in Adolescence | p=.40 ; would be included when smoking and marijuana removed instead |

**Table 10**

*Pre and Post-LASSO Model Comparison*

| Variable | Flourishing | | | | Psychological Distress | | | | Economic Worry | | | |
| --- | --- | --- | --- | --- | --- | --- | --- | --- | --- | --- | --- | --- |
|  | Model 1) | | Model 2) | | Model 1) | | Model 2) | | Model 1) | | Model 2) | |
|  | Pre-LASSO | Post-LASSO | Pre-LASSO | Post-LASSO | Pre-LASSO | Post-LASSO | Pre-LASSO | Post-LASSO | Pre-LASSO | Post-LASSO | Pre-LASSO | Post-LASSO |
| **Economic** |  |  |  |  |  |  |  |  |  |  |  |  |
| Food Secure | 0.168*  (0.097) | 0.168*  (0.098) | 0.140  (0.098) | 0.140  (0.098) | -0.167*  (0.085) | -0.166*  (0.084) | -0.148*  (0.087) | -0.127  (0.084) | -0.229**  (0.087) | -0.228**  (0.087) | -0.182**  (0.090) | -0.179**  (0.090) |
|  |  |  |  |  |  |  |  |  |  |  |  |  |
| **Education** |  |  |  |  |  |  |  |  |  |  |  |  |
| Attended Preschool | -- | -- | -- | -- | -0.106**  (0.044) | -0.102**  (0.045) | -0.112**  (0.046) | -0.110**  (0.045) | -- | -- | -- | -- |
| Graduated High School on Time | 0.282***  (0.075) | 0.274***  (0.074) | 0.265***  (0.074) | 0.265***  (0.074) | -0.218**  (0.085) | -0.224**  (0.086) | 0.223**  (0.088) | -0.210**  (0.087) | -0.352***  (0.081) | -0.357***  (0.080) | -0.328***  (0.084) | -0.333***  (0.083) |
| Math Proficiency | -- | -- | -- | -- | -- | -- | -- | -- | -0.114*  (0.064) | -0.121**  (0.058) | -0.102*  (0.059) | -0.105*  (0.058) |
| Reading Proficiency | 0.139**  (0.061) | 0.163***  (0.050) | 0.150***  (0.051) | 0.150***  (0.051) | -0.067  (0.046) | -0.067  (0.046) | -- | -- | -- | -- | -- | -- |
|  |  |  |  |  |  |  |  |  |  |  |  |  |
| **Health** |  |  |  |  |  |  |  |  |  |  |  |  |
| Non-Low Birthweight | 0.125**  (0.056) | 0.123**  (0.055) | 0.111**  (0.054) | 0.111**  (0.054) | -- | -- | -- | -- | -0.097*  (0.055) | -0.096*  (0.053) | -0.086  (0.053) | -0.078  (0.052) |
| Non-Obese | -- | -- | -- | -- | -- | -- | -- | -- | -0.084*  (0.047) | -0.084*  (0.046) | -0.065  (0.045) | -0.066  (0.045) |
| Not in Fair/Poor General Health | -- | -- | -- | -- | -- | -- | -- | -- | -- | -- | -- | -- |
|  |  |  |  |  |  |  |  |  |  |  |  |  |
| **Health Behaviors** |  |  |  |  |  |  |  |  |  |  |  |  |
| Didn’t Smoke Regularly in Adolescence | 0.222***  (0.075) | 0.236***  (0.069) | 0.239***  (0.068) | 0.239***  (0.068) | -0.234***  (0.067) | -0.224***  (0.065) | -0.247***  (0.062) | -0.238***  (0.063) | -0.244***  (0.060) | -0.267***  (0.055) | -0.255***  (0.059) | -0.267***  (0.054) |
| Didn’t Try Marijuana in Adolescence | -- | -- | -- | -- | -0.150**  (0.063) | -0.130**  (0.061) | -0.110*  (0.057) | -0.107*  (0.058) | -- | -- | -- | -- |
|  |  |  |  |  |  |  |  |  |  |  |  |  |
| **Family, Peers, Community** |  |  |  |  |  |  |  |  |  |  |  |  |
| Never Arrested | -- | -- | -- | -- | -- | -- | -- | -- | -- | -- | -- | -- |
|  |  |  |  |  |  |  |  |  |  |  |  |  |
| **Economic - Contextual** | - | - |  |  | - | - |  |  | - | - |  |  |
| Household Never Housing Burdened | - | - | 0.149***  (0.047) | 0.149***  (0.047) | - | - | -0.164***  (0.055) | -0.152***  (0.055) | - | - | -0.195***  (0.053) | -0.199***  (0.052) |
| Household Never in Poverty | - | - | -- | -- | - | - | -- | -- | - | - | -- | -- |
|  |  |  |  |  |  |  |  |  |  |  |  |  |
| **Family, Peers, Community - Contextual** | - | - |  |  | - | - |  |  | - | - |  |  |
| Household Head Never Unmarried | - | - | -- | -- | - | - | 0.176***  (0.055) | 0.131**  (0.052) | - | - | -0.095*  (0.050) | -0.105*  (0.050) |
|  |  |  |  |  |  |  |  |  |  |  |  |  |
| R-Squared | 0.059 | 0.057 | 0.062 | 0.062 | 0.050 | 0.049 | 0.061 | 0.058 | 0.075 | 0.075 | 0.088 | 0.088 |

**Table 10**

*Pre and Post-LASSO Model Comparison*

| Variable | Social Anxiety | | | | Life Satisfaction | | | | |
| --- | --- | --- | --- | --- | --- | --- | --- | --- | --- |
|  | Model 1) | | Model 2) | | Model 1) | | Model 2) | | |
|  | Pre-LASSO | Post-LASSO | Pre-LASSO | Post-LASSO | Pre-LASSO | Post-LASSO | Pre-LASSO | Post-LASSO |  |
| **Economic** |  |  |  |  |  |  |  |  |  |
| Food Secure | -0.185**  (0.080) | -0.177**  (0.082) | -0.172**  (0.081) | -0.148*  (0.084) | 0.170*  (0.094) | 0.171*  (0.094) | 0.137  (0.096) | 0.138  (0.096) |  |
|  |  |  |  |  |  |  |  |  |  |
| **Education** |  |  |  |  |  |  |  |  |  |
| Attended Preschool | -- | -- | -- | -- | -- | -- | -- | -- |  |
| Graduated High School on Time | -0.231**  (0.093) | -0.171**  (0.079) | -0.223**  (0.090) | -0.157*  (0.080) | 0.274***  (0.063) | 0.273***  (0.063) | 0.264***  (0.064) | 0.262***  (0.064) |  |
| Math Proficiency | -0.095  (0.078) | -0.118*  (0.067) | -0.085  (0.076) | -0.107  (0.066) | -- | -- | -- | -- |  |
| Reading Proficiency | -- | -- | -- | -- | -- | -- | -- | -- |  |
|  |  |  |  |  |  |  |  |  |  |
| **Health** |  |  |  |  |  |  |  |  |  |
| Non-Low Birthweight | -- | -- | -- | -- | -- | -- | -- | -- |  |
| Non-Obese | -- | -- | -- | -- | -- | -- | -- | -- |  |
| Not in Fair/Poor General Health | -0.163  (0.113) | -0.136  (0.110) | -- | -- | -- | -- | -- | -- |  |
|  |  |  |  |  |  |  |  |  |  |
| **Health Behaviors** |  |  |  |  |  |  |  |  |  |
| Didn’t Smoke Regularly in Adolescence | -- | -- | -- | -- | 0.100*  (0.054) | 0.104*  (0.053) | 0.106**  (0.053) | 0.112**  (0.052) |  |
| Didn’t Try Marijuana in Adolescence | -- | -- | -- | -- | 0.184***  (0.048) | 0.192***  (0.047) | 0.173***  (0.047) | 0.183***  (0.052) |  |
|  |  |  |  |  |  |  |  |  |  |
| **Family, Peers, Community** |  |  |  |  |  |  |  |  |  |
| Never Arrested | -- | -- | -- | -- | 0.139*  (0.080) | 0.141*  (0.079) | 0.134*  (0.079) | 0.136*  (0.079) |  |
|  |  |  |  |  |  |  |  |  |  |
| **Economic - Contextual** | - | - |  |  | - | - |  |  |  |
| Household Never Housing Burdened | - | - | -- | -- | - | - | 0.166***  (0.052) | 0.164***  (0.052) |  |
| Household Never in Poverty | - | - | -0.138**  (0.060) | -0.101*  (0.055) | - | - | -- | -- |  |
|  |  |  |  |  |  |  |  |  |  |
| **Family, Peers, Community - Contextual** | - | - |  |  | - | - |  |  |  |
| Household Head Never Unmarried | - | - | -- | -- | - | - | -- | -- |  |
|  |  |  |  |  |  |  |  |  |  |
| R-Squared | 0.051 | 0.039 | 0.056 | 0.040 | 0.082 | 0.082 | 0.089 | 0.089 |  |

Notes: Model 1) includes individual-level measures only ; Model 2) includes individual and contextual-level measures.

*(**)[***] indicates estimate is statistically significant at the .1(.05)[.01] level.

“-“ indicates measure not included in regressions. “--” indicates covariate not included in Post-LASSO specification. These estimates are suppressed in the output for the pre-LASSO models for ease of interpretation.

**Figure 3**

*Distribution of Highest Earnings During Ages 20-29*


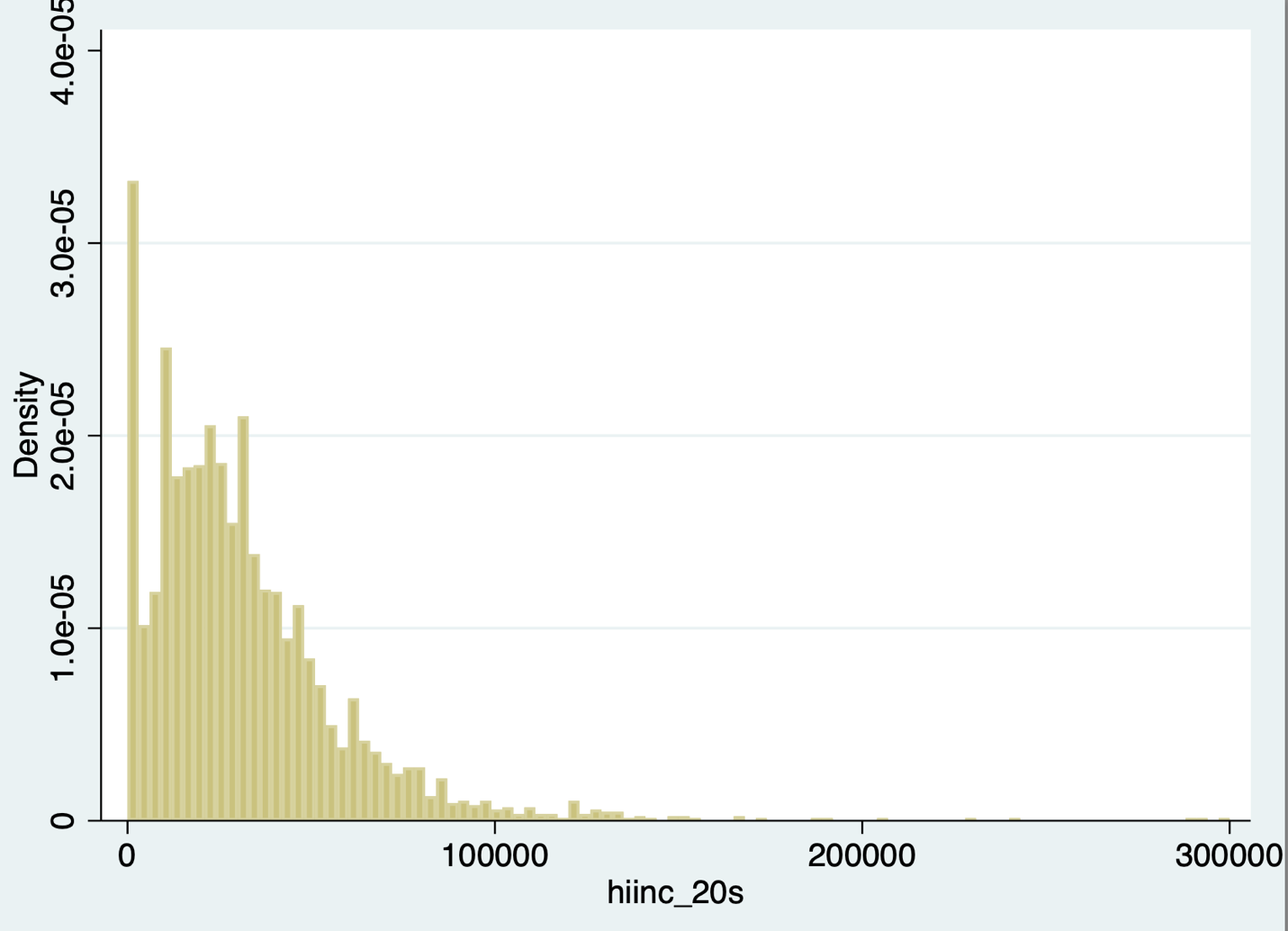


Notes: N=2,889. Measure is constructed using the following information in the 2004-2018 waves, which alternative every other year: labor earnings (collected for head, spouse, and other members separately), farming earnings (collected for head and spouse together), business earnings (collected for head and spouse together). Additional variables on household relationship to head and whether farming / business earnings belonged to one member are used to assign earnings to the individual respondent. Earnings are inflation-adjusted to 2018 dollars.

**Table 11**

*Annie E. Casey KIDS COUNT Index and Data Sources*

| **Domain** | **Measure** | **State-Level Source** |
| --- | --- | --- |
| Health | Percent of babies Born with Low Birthweight (less than 5.5 pounds) | CDC, NCHS, Vital Statistics |
|  | Percent of children Under 19 Without Health Insurance | U.S. Census Bureau, ACS |
|  | Child and teen Deaths (age 1-19) per 1,000 ^1^ | CDC, NCHS, Vital Statistics |
|  | Percent of children (12-19) reporting dependence or abuse of alcohol or illicit drugs ^2^ | SAMHSA, NSDUH. |
|  |  |  |
| Education | Percent of young children (3-4) not enrolled in school in the previous three months | U.S. Census Bureau, ACS |
|  | Percentage of 4th grade public school students not proficient in reading ^3^ | USDE-NCES, NAEP |
|  | Percentage of 8th grade public school students not proficient in math ^3^ | USDE-NCES, NAEP |
|  | Percentage of entering freshman class not graduating on time | USDE-NCES, Common Core of Data. |
|  |  |  |
| Family and Community | Percent of children (Under 18) living with their own unmarried parents | U.S. Census Bureau, ACS |
|  | Percent of children (Under 18) living in a household where the household head lacks a high school diploma or equivalent degree | U.S. Census Bureau, ACS |
|  | Percent of children (Under 18) living in high poverty areas (census tracts with poverty rates of 30% or more) ^4^ | U.S. Census Bureau, ACS |
|  | Teen Births (Females 15-19) per 1,000 | CDC, NCHS, Vital Statistics |
|  |  |  |
| Economic Well-Being | Percent of children (Under 18) in poverty | U.S. Census Bureau, ACS |
|  | Percent of children (Under 18) in families without secure employment (no parent has regular, full-time, year-round employment) | U.S. Census Bureau, ACS |
|  | Percent of children (Under 18) in families with a high housing cost burden (more than 30% of monthly household pretax income is spent on housing) | U.S. Census Bureau, ACS |
|  | Percent of teenagers (16-19) not in school and not working ^5^ | U.S. Census Bureau, ACS |
| **Source**: The Annie E. Casey Foundation. (2019). 2019 KIDS COUNT Data Book: State Trends in Child Wellbeing. Retrieved from Baltimore, MD: https://www.aecf.org/m/resourcedoc/aecf-2019kidscountdatabook-2019.pdf | | |
| **Notes**: CDC = Centers for Disease Control and Prevention ; NCHS = National Center for Health Statistics ; ACS = American Community Survey ; SAMHSA = Substance Abuse and Mental Health Services Administration ; NSDUH = National Survey on Drug Use and Health ; USDE-NCES = U.S. Department of Education, National Center for Education Statistics **;** NAEP **=** National Assessment of Educational Progress.  The measures here are the latest measures and data sources for the KIDS COUNT index. We construct an individual-level index that mirrors the KIDS COUNT methodology using information on the PSID. The notes below denote the alterations required to construct the index.  ^1^ Individual-level index replaces mortality data with general health status  ^2^ Individual-level index separates these into drank regularly and tried marijuana  ^3^ Individual-level index does not use age-specific test scores  ^4^ Census tract indicators not available on individual-level PSID data  ^5^ Sample size in PSID is too small to reliably estimate (n<5). | | |

**Table 12**

*Final Weights For Alternate Specifications of Index*

| Variable | Index Weight | | | | | | | |
| --- | --- | --- | --- | --- | --- | --- | --- | --- |
|  | Preferred Specification | | Full Model | | Pure Post-LASSO | | Full Model, Pure Post -LASSO | |
|  | 1) | 2) | 1) | 2) | 1) | 2) | 1) | 2) |
| **Economic** |  |  |  |  |  |  |  |  |
| Food Secure | 0.199 | 0.145 | 0.191 | 0.158 | 0.206 | 0.154 | 0210 | 0.185 |
|  |  |  |  |  |  |  |  |  |
| **Education** |  |  |  |  |  |  |  |  |
| Attended Preschool | 0.025 | 0.024 | 0.019 | 0.023 | 0.025 | 0.024 | 0.020 | 0.021 |
| Graduated High School on Time | 0.285 | 0.245 | 0.283 | 0.261 | 0.303 | 0.258 | 0.319 | 0.300 |
| Math Proficiency | 0.052 | 0.040 | 0.040 | 0.036 | 0.060 | 0.036 | 0.048 | 0.044 |
| Reading Proficiency | 0.054 | 0.031 | 0.073 | 0.061 | 0.068 | 0.042 | 0.084 | 0.075 |
|  |  |  |  |  |  |  |  |  |
| **Health** |  |  |  |  |  |  |  |  |
| Non-Low Birthweight | 0.052 | 0.040 | 0.066 | 0.052 | 0.053 | 0.042 | 0.068 | 0.054 |
| Non-Obese | 0.020 | 0.013 | 0.028 | 0.018 | 0.023 | 0.013 | 0.028 | 0.016 |
| Not in Fair/Poor General Health | 0.027 | - | 0.080 | - | 0.053 | 0.024 | 0.086 | 0.068 |
|  |  |  |  |  |  |  |  |  |
| **Health Behaviors** |  |  |  |  |  |  |  |  |
| Didn’t Smoke Regularly in Adolescence | 0.192 | 0.178 | 0.203 | 0.205 | 0.216 | 0.203 | 0.228 | 0.222 |
| Didn’t Drink Regularly in Adolescence | - | - | - | - | -0.020 | 0.009 | -0.038 | -0.023 |
| Didn’t Try Marijuana in Adolescence | 0.069 | 0.056 | 0.066 | 0.047 | 0.075 | 0.038 | 0.084 | 0.063 |
| Never Pregnant | - | - | - | - | -0.015 | -0.016 | -0.094 | -0.091 |
|  |  |  |  |  |  |  |  |  |
| **Family, Peers, Community** |  |  |  |  |  |  |  |  |
| Never Arrested | 0.027 | 0.022 | -0.050 | -0.050 | -0.045 | -0.018 | -0.043 | -0.044 |
|  |  |  |  |  |  |  |  |  |
| **Contextual** | - |  | - |  |  |  |  |  |
| Household Never Housing Burdened | - | 0.136 | - | 0.137 | - | 0.138 | - | 0.148 |
| Household Never in Poverty | - | 0.018 | - | -0.023 | - | 0.024 | - | 0.009 |
| Parents Never Unemployed | - | - | - | - | - | -0.049 | - | -0.061 |
| Never Moved | - | - | - | - | - | 0.013 | - | -0.014 |
| Never in Household Where Parents without HS Degree | - | - | - | - | - | 0.002 | - | -0.080 |
| Household Head Never Unmarried | - | 0.051 | - | 0.074 | - | 0.060 | - | 0.108 |

Notes: Estimates here are constructed by the following procedure: 1) multiply the absolute value of the product of the regression estimates for each subjective outcome presented in Table 3 with its respective PCA coefficient from Component 1 in Table 2 ; 2) averaging across the 5 outcomes ; and 3) multiplying the vector of weights by a scalar so that each set sums to 1. Underlying logistic regression models control for age, sex, and race/ethnicity. Model 1) contains only individual covariates ; Model 2) contains individual and contextual covariates. “Full model” refers to models that do not parse covariates if they appear in at least one subjective outcome model – i.e. if low birthweight is kept in at least one subjective outcome model, it is included as a covariate for all five models in the final weight regression. “Post-LASSO” models only adopt model selection via adaptive LASSO: there is no post hoc backwards selection procedure.

**Table 13**

*Marginal Effect and Partial R^2^ of Increased Index Score on Young Adulthood Outcomes*

| Index Type | Specification | Statistic | Ever in Fair / Poor Health | Ever Depressed 2 Weeks in Past Year^1^ | Highest Earnings Between Ages 20-29^3^ |
| --- | --- | --- | --- | --- | --- |
| Child and Adolescent Thriving Index 1.0 – Individual | Preferred | Marginal Effect | -0.079  (-0.060 – -0.098) | -0.063  (-0.046 – -0.080) | 0.172  (0.137 – 0.206) |
|  |  | Partial R^2^ | 0.029  (0.045 vs. 0.016) | 0.019  (0.033 vs. 0.014) | 0.042  (0.312 vs. 0.270) |
|  | Full | Marginal Effect | -0.079  (-0.060 – -0.098) | -0.062  (-0.045 – -0.079) | 0.170  (0.137 – 0.202) |
|  |  | Partial R^2^ | 0.030  (0.046 vs. 0.016) | 0.018  (0.032 vs. 0.014) | 0.041  (0.311 vs. 0.270) |
|  | Post-LASSO | Marginal Effect | -0.077  (-0.058 – -0.096) | -0.062  (-0.045 – -0.079) | 0.169  (0.135 – 0.201) |
|  |  | Partial R^2^ | 0.029  (0.045 vs. 0.016) | 0.018  (0.032 vs. 0.014) | 0.047  (0.317 vs. 0.270) |
|  | Full & Post-LASSO | Marginal Effect | -0.077  (-0.058 – -0.096) | -0.062  (-0.045 – -0.079) | 0.156  (0.122 – 0.189) |
|  |  | Partial R^2^ | 0.029  (0.045 vs. 0.016) | 0.017  (0.031 vs. 0.014) | 0.045  (0.315 vs. 0.270) |
|  |  |  |  |  |  |
| Child and Adolescent Thriving Index 1.0 – Individual and Contextual | Preferred | Marginal Effect | -0.084  (-0.065 – -0.103) | -0.068  (-0.051 – -0.085) | 0.180  (0.148 – 0.212) |
|  |  | Partial R^2^ | 0.035  (0.051 vs. 0.016) | 0.023  (0.037 vs. 0.014) | 0.047  (0.317 vs. 0.270) |
|  | Full | Marginal Effect | -0.084  (-0.065 – -0.103) | -0.066  (-0.049 – -0.083) | 0.173  (0.142 – 0.204) |
|  |  | Partial R^2^ | 0.036  (0.052 vs. 0.016) | 0.022  (0.036 vs. 0.014) | 0.045  (0.315 vs. 0.270) |
|  | Post-LASSO | Marginal Effect | -0.085  (-0.066 – -0.104) | -0.066  (-0.049 – -0.083) | 0.172  (0.139 – 0.203) |
|  |  | Partial R^2^ | 0.036  (0.052 vs. 0.016) | 0.022  (0.036 vs. 0.014) | 0.044  (0.314 vs. 0.270) |
|  | Full & Post-LASSO | Marginal Effect | -0.085  (-0.065 – -0.105) | -0.064  (-0.046 – -0.082) | 0.153  (0.120 – 0.184) |
|  |  | Partial R^2^ | 0.035  (0.051 vs. 0.016) | 0.020  (0.034 vs. 0.014) | 0.035  (0.305 vs. 0.270) |
|  |  |  |  |  |  |
| KIDS COUNT Reconstructed | N/A | Marginal Effect | -0.075  (-0.055 – -0.095) | -0.057  (-0.040 – -0.074) | 0.177  (0.144 – 0.209) |
|  |  | Partial R^2^ | 0.026  (0.042 vs. 0.016 | 0.015  (0.029 vs. 0.014) | 0.044  (0.314 vs. 0.270) |

Notes: Marginal effect estimates show effect of a one standard deviation increase of index score on later health, with 95% Confidence Interval in parentheses. Marginal effect estimates use *mimrgns* user-written command in STATA, which estimates predictive margins accommodating for multiple imputation. Underlying logistic regression models control for age, sex, and race/ethnicity.

Partial R^2^ shows the increase in R^2^ when adding the index score to a model controlling for demographic factors, with the respective R^2^ for each model shown in parentheses.

Reweighted health status is a variable that is constructed by recoding the 5 point general health measure to a semi-continuous measure using results from a prior study of Canadians 12 and older. (Van Doorslaer & Jones, 2003)

Refer to Appendix Table 7 for candidate components in the “Individual”, “Individual and Contextual” , and “Individual, Contextual, and Additional” indices. Refer to Appendix Table 1 for candidate components in the KIDS COUNT index.

**Table 14**

*Comparison when Using Subjective Measures from Childhood and Adolescence*

| Variable | Flourishing | | | | Psychological Distress | | | |
| --- | --- | --- | --- | --- | --- | --- | --- | --- |
|  | Model 1) | | Model 2) | | Model 1) | | Model 2) | |
| Time Frame Collected | Young Adult | Child/ Adolescent | Young Adult | Child/ Adolescent | Young Adult | Child/ Adolescent | Young Adult | Child/ Adolescent |
| **Economic** |  |  |  |  |  |  |  |  |
| Food Secure | 0.168*  (0.098) | 0.209**  (0.104) | 0.140  (0.098) | 0.209**  (0.104) | -0.166*  (0.084) | -- | -0.127  (0.084) | -- |
|  |  |  |  |  |  |  |  |  |
| **Education** |  |  |  |  |  |  |  |  |
| Attended Preschool | -- | -- | -- | -- | -0.102**  (0.045) | -0.103  (0.064) | -0.110**  (0.045) | -0.103  (0.064) |
| Graduated High School on Time | 0.274***  (0.074) | 0.339***  (0.099) | 0.265***  (0.074) | 0.339***  (0.099) | -0.224**  (0.086) | -0.268***  (0.114) | -0.210**  (0.087) | -0.268***  (0.114) |
| Math Proficiency | -- | 0.177***  (0.068) | -- | 0.177***  (0.068) | -- | -- | -- | -- |
| Reading Proficiency | 0.163***  (0.050) | -- | 0.150***  (0.051) | -- | -0.067  (0.046) | -- | -- | -- |
|  |  |  |  |  |  |  |  |  |
| **Health** |  |  |  |  |  |  |  |  |
| Non-Low Birthweight | 0.123***  (0.055) | -- | 0.111**  (0.054) | -- | -- | -- | -- | -- |
| Non-Obese | -- | -- | -- | -- | -- | -- | -- | -- |
| Not in Fair/Poor General Health | -- | -- | -- | -- | -- | -- | -- | -- |
|  |  |  |  |  |  |  |  |  |
| **Health Behaviors** |  |  |  |  |  |  |  |  |
| Didn’t Smoke Regularly in Adolescence | 0.236***  (0.069) | 0.223***  (0.066) | 0.239***  (0.068) | 0.223***  (0.066) | -0.224***  (0.065) | -0.306***  (0.076) | -0.238***  (0.063) | -0.306***  (0.076) |
| Didn’t Drink Regularly in Adolescence | -- | 0.114  (0.077) | -- | 0.114  (0.077) | -- | -0.228**  (0.099) | -- | -0.228**  (0.099) |
| Didn’t Try Marijuana in Adolescence | -- | -- | -- | -- | -0.130**  (0.061) | -- | -0.107*  (0.058) | -- |
| Never Pregnant | -- | -- | -- | -- | -- | -- | -- | -- |
|  |  |  |  |  | -- | -- | -- | -- |
| **Family, Peers, Community** |  |  |  |  |  |  |  |  |
| Never Arrested | -- | 0.206**  (0.092) | -- | 0.206**  (0.092) | - | -0.209  (0.130) | -- | -0.209  (0.130) |
|  |  |  |  |  |  |  |  |  |
| **Economic - Contextual** | - | - |  |  | - | - |  |  |
| Household Never Housing Burdened | - | - | 0.149***  (0.047) | -- | - | - | -0.152***  (0.055) | -- |
| Never in Household Poverty | - | - | -- | -- | - | - | -- | -- |
| Parent Never Unemployed | - | - | -- | -- | - | - | -- | -- |
|  |  |  |  |  |  |  |  |  |
| **Family, Peers, Community - Contextual** | - | - |  |  | - | - |  |  |
| Never Moved | - | - | -- | -- | - | - | -- | -- |
| Never in Household with no Parent With HS Degree | - | - | -- | -- | - | - | -- | -- |
| Never in Household with Unmarried Head | - | - | -- | -- | - | - | -0.131**  (0.052) | -- |

Notes: Model 1) includes individual-level measures only ; Model 2) includes individual and contextual-level measures.

“-“ indicates measure not included in initial regressions. “--” indicates covariate not included post-model selection (LASSO and post-LASSO).
